# Hydroxylated Rh Single‐Atom Antennas Assembled on Carbon Nitride Toward Stable Photocatalytic Hydrogen Evolution

**DOI:** 10.1002/advs.202518847

**Published:** 2025-12-16

**Authors:** Chunmei Li, Pingfan Zhang, Ming Zheng, Shasha Cheng, Baodong Mao, Guangbo Che, Song Wang, Weidong Shi, Hongjun Dong

**Affiliations:** ^1^ Institute of Green Chemistry and Chemical Technology School of Chemistry and Chemical Engineering Jiangsu University Zhenjiang 212013 P. R. China; ^2^ MIIT Key Laboratory of Critical Materials Technology for New Energy Conversion and Storage School of Chemistry and Chemical Engineering Harbin Institute of Technology Harbin 150001 P. R. China; ^3^ Jilin Provincial Key Laboratory of Western Jilin's Clean Energy College of Chemistry Baicheng Normal University Baicheng 137000 P. R. China; ^4^ Hubei Key Laboratory of Low Dimensional Optoelectronic Materials and Devices Hubei University of Arts and Science Xiangyang 441053 P. R. China

**Keywords:** charge transfer kinetics, dual‐cycle path, hydroxylated Rh‐SAAs, photocatalytic hydrogen evolution, single‐atom catalyst

## Abstract

Polymeric carbon nitride (PCN)‐based single‐atom catalysts represent the most promising catalysts for photocatalytic hydrogen evolution (PHE), which, however, still suffer from reduced thermodynamic stability because of the metal‐induced heptazine skeleton distortion. Herein, hydroxylated Rh single‐atom antennas (Rh‐SAAs) connected by dual oxygen‐bridges are constructed on the surface of PCN matrix, which brings great structural advantages in avoiding skeleton distortion and increasing stability compared to the traditional direct coordination of metal atoms onto PCN. The optimal PCN‐Rh‐0.5 delivers an average PHE rate of 3409 µmol g^−1^ h^−1^, 32.5 times that of the PCN/Pt benchmark. More importantly, it achieves an ultralong stable operation time (192 h) with a higher amount of hydrogen production per unit mass than those of the state‐of‐the‐art single‐atom catalysts and other high‐stability catalysts (≥50 h) under the same conditions. Insights into the mechanism reveal the key role of the electron pump effect induced by interband trap states composed of hybridized Rh 4d/O 2p orbitals that can propel the directed electron transfer toward Rh‐SAAs. As a result, the improved charge separation efficiency and lifetime, along with the strong protonation capability with dual oxygen‐bridges, trigger a dual‐cycle reaction path, thereby achieving high PHE activity and stability.

## Graphical abstract

PCN‐Rh single‐atom catalyst shows a dual‐cycle reaction path for photocatalytic hydrogen evolution (PHE). PCN‐Rh‐0.5 achieves 32.5 times the PHE rate of PCN/Pt, which exhibits a longer operation time (192 h) with a higher amount of hydrogen production (654.5 mmol g^−1^) than the reported PCN‐based single‐atom catalysts and other high‐stability catalysts with operation time ≥50 h under the same conditions.

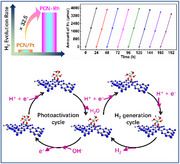

## 1 Introduction

Hydrogen is expected to be a main clean energy carrier to replace traditional fossil fuels in the future.^[^ ^1^, ^2^ ^]^ Solar‐driven photocatalytic hydrogen evolution (PHE) from water splitting has been deemed a promising green hydrogen production technology, because of the direct solar‐to‐hydrogen conversion without additional energy consumption.^[^ ^3^, ^4^, ^5^ ^]^ However, the low energy conversion efficiency, slow hydrogen production, and poor stability of the photocatalysts have long been the hindrances to the advancement of PHE technology to industrial application. Hence, developing advanced strategies to confront these issues is significantly anticipated, but still remains a huge challenge due to the undesired structural variation of the actual catalytic sites induced by light irradiation and catalysis during photocatalysis.

Polymeric carbon nitride (PCN), as a seductively cost‐efficient polymer photocatalyst, demonstrates great potential in various photocatalytic fields.^[^ ^6^, ^7^ ^]^ For PHE applications, tremendous efforts have been dedicated to exploiting effective strategies to improve the comprehensive performance of PCN, including the surface decoration, electronic structure regulation, molecular skeleton modification, defect engineering, supramolecular self‐assembly, et al.^[^ ^8^, ^9^, ^10^, ^11^, ^12^ ^]^ Single‐atom catalysts have stood out from the modified PCN composites owing to their high atomic utilization, abundant active sites, and catalytic selectivity.^[^ ^13^, ^14^, ^15^, ^16^ ^]^ Specifically, the defined but adjustable coordination configuration of single metal atoms immobilized on the PCN skeleton allows for a more profound and accurate understanding of the reaction mechanism and charge transfer kinetics at the atomic level, which is crucial to deliver excellent PHE performance through the directed design of metal atom configuration.^[^ ^17^, ^18^, ^19^ ^]^ For example, Shi et al. constructed partially oxidized Ni single‐atom sites on PCN, which produce abundant unpaired electrons in 3d orbits that can be photoexcited, thereby increasing the PHE activity by more than 30 times.^[^ ^17^ ^]^ Another study presents the design of dual coordination configurations of Cu‐N_3_ on PCN monolayer and Cu‐N_4_ between two PCN layers, the assembled mode of which dramatically promotes the in‐plane and interlayer separation/transfer of photogenerated charge carriers, achieving a 30‐fold PHE rate of pure PCN.^[^ ^18^ ^]^ However, in most works, the distorted skeleton of PCN originated from the intralayer/interlayer coordination of metal atoms often leads to reduced thermodynamic stability, which leads to the difficulty for PHE to achieve long‐term operation with high efficiency.

In this contribution, we fabricate hydroxylated Rh single‐atom antennas (Rh‐SAAs) connected by dual oxygen‐bridges on the surface of PCN matrix, which provides the superior structure rigidity and prevents the skeleton distortion to the greatest extent to enhance the structural stability. This unique antenna configuration is confirmed by X‐ray photoelectron spectroscopy (XPS) and X‐ray absorption fine structure (XAFS) combined with density functional theory (DFT) calculations. As certified by the femto‐second transient absorption (fs‐TA) spectra, time‐resolved photoluminescence (TR‐PL) decay curves, and in situ XPS, a trap‐state‐induced electron pump effect of hydroxylated Rh‐SAAs was revealed, which dramatically improves the separation efficiency and lifetime of the photogenerated charge carriers. As expected, the optimal PCN‐Rh‐0.5 delivers a high PHE rate of 32.5 times that of the PCN/Pt benchmark and an outstanding stable operation time of up to 192 h.

## 2 Results and Discussion

**Figure 1:**
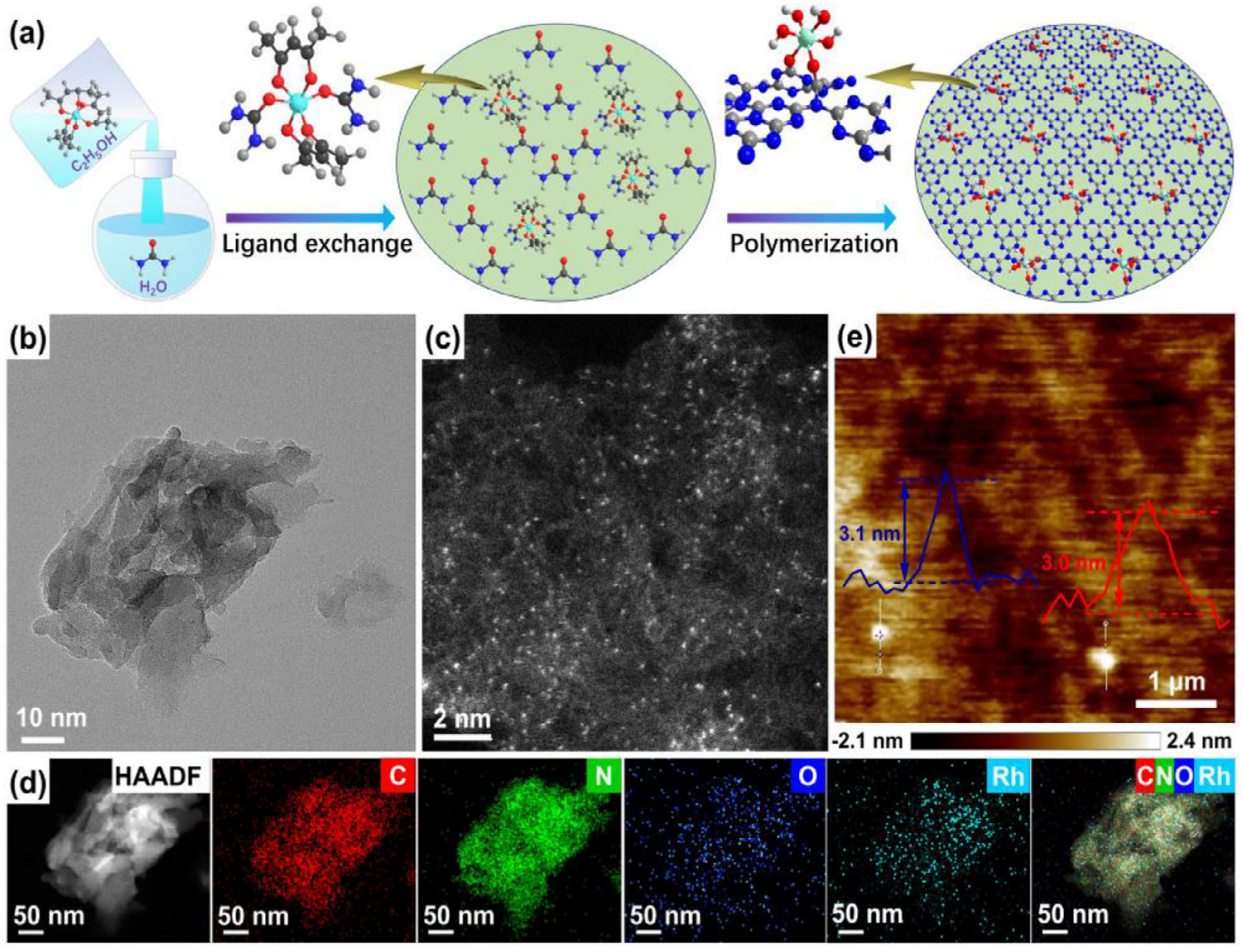
a) Schematic synthesis process. b) TEM, c) SAC HAADF‐TEM, d) HAADF‐TEM and EDS elemental mappings, and e) AFM (inset: thickness profile) images of PCN‐Rh‐0.5.

The fabrication process, employing a magical ligand exchange and polymerization (LEP) strategy, for the hydroxylated Rh‐SAAs on the surface of the PCN matrix is depicted in **Figure** **1**a. When the ethanol solution of rhodium (III) acetylacetone is slowly added dropwise to the aqueous solution of urea under strong stirring, the urea molecules are coordinated with the Rh atom through ligand exchange to improve the dissolvability and dispersion of Rh species in solution, owing to the microsolubility of rhodium (III) acetylacetone in water, thus forming an atomically dispersed Rh precursor. Subsequently, the hydroxylated Rh‐SAAs connected by dual oxygen‐bridges are assembled on the surface of the PCN matrix through a pyrolysis of the Rh precursor in the thermal polymerization of urea. The transmission electron microscopy (TEM) image of PCN‐Rh‐0.5 in Figure 1b shows a typical 2D nanostructure. The spherical aberration‐corrected high‐angle annular dark field‐TEM (SAC HAADF‐TEM) image of PCN‐Rh‐0.5 displays an atomically dispersed Rh distribution and mesoporous on the PCN matrix (Figure 1c). The nitrogen adsorption–desorption experiments also indicate the existence of mesoporous in PCN‐Rh‐0.5 (Figures S1 and S2 and Table S1, Supporting Information). The HAADF‐TEM and energy‐dispersive spectroscopy (EDS) elemental mappings demonstrate the introduction of Rh and O atoms into the skeleton of PCN owing to the uniform distribution of C, N, O, and Rh elements throughout the nanosheet (Figure 1d). Additionally, the atomic force microscope (AFM) image in Figure 1e depicts that the thickness of ≈3 nm for the PCN‐Rh‐0.5 nanosheet is less than half that of PCN (Figure S3, Supporting Information), which can effectively reduce the carrier recombination probability in the (001) crystal plane direction by shortening the charge migration path. Moreover, the X‐ray diffraction (XRD) patterns of all PCN‐Rh samples (Figure S4, Supporting Information) include (002) peak assigned to the interlayer stacking of the aromatic rings and (100) peak attributed to the repetitive structural packing of tri‐s‐triazine heterocycles in the skeleton of PCN, without diffraction peaks from Rh metal and Rh oxides.^[^ ^20^ ^]^ In addition, the Fourier transform infrared (FT‐IR) spectra of all PCN‐Rh samples exhibit the same fingerprint peaks as PCN, including typical stretching vibration of heterocycles at 1000–1700 cm^−1^ and breathing vibration of heptazine units at 815 cm^−1^ (Figure S5, Supporting Information).^[^ ^21^ ^]^ Noticeably, a weak broad absorption appears between 3790 and 3900 cm^−1^ for the PCN‐Rh samples (Figure S6, Supporting Information), which may result from Rh‐OH groups in the hydroxylated Rh‐SAAs because of the increased oxygen content in them relative to PCN (Table S2, Supporting Information).

**Figure 2:**
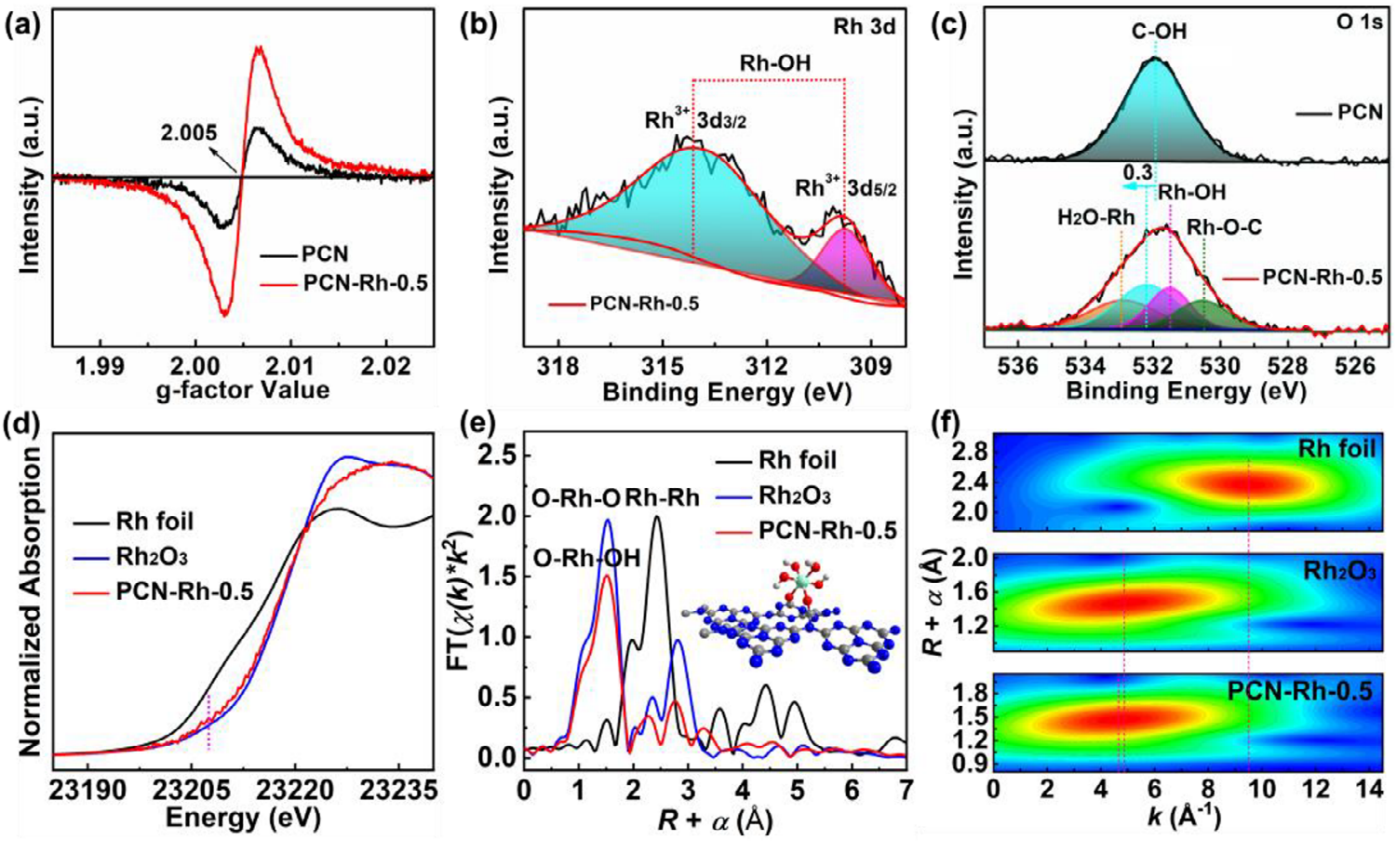
a) EPR spectra, b) Rh 3d XPS, c) O 1s XPS, d) Rh *K*‐edge XANES, e) FT‐EXAFS, and f) wavelet transforms for EXAFS of different samples.

**Figure** **2**a shows electron paramagnetic resonance (EPR) spectra. The weak signal for PCN originates from the intrinsic nitrogen defect, while the enhanced one for PCN‐Rh‐0.5 certifies the increase of nitrogen defects owing to the surface modification of hydroxylated Rh‐SAAs. It is also reflected by the significantly increased population of mesoporous (Figure S2, Supporting Information) and decreased nitrogen content in PCN‐Rh‐0.5 relative to PCN (Table S2, Supporting Information). For PCN, the photogenerated electrons captured by the trap level tend to recombine with photogenerated holes owing to the short transfer distance in the isolated intrinsic defect region, resulting in a short carrier lifetime.^[^ ^22^ ^]^ In comparison, due to the drastically increased nitrogen defect, PCN‐Rh‐0.5 may produce a continuous defect region to provide a sufficiently long transfer path for the photogenerated electrons and then inhibit recombination with photogenerated holes, thus improving lifetime and separation efficiency of carriers.^[^ ^22^ ^]^ The X‐ray photoelectron spectra (XPS) are used to ascertain the chemical state of elements. Notably, the slight shift to high binding energy of C 1s and N 1s XPS is produced on PCN‐Rh‐0.5 in comparison with PCN (Figures S7–S9, Supporting Information), which is because the strong electron‐withdrawing effect of hydroxylated Rh‐SAAs leads to reduced electron cloud density of the PCN skeleton. The Rh 3d XPS of PCN‐Rh‐0.5 in Figure 2b shows a pair of spin‐orbit coupling peaks at 309.8 and 314.1 eV, which are well consistent with those of Rh(OH)_3_.^[^ ^23^ ^]^ indicating the formation of hydroxylated Rh‐SAAs on the surface of PCN matrix. Further surface bonding information of Rh atoms on PCN is revealed by O 1s XPS, as displayed in Figure 2c. A symmetric binding energy peak arises from hydroxyl groups on the PCN skeleton in the O 1s XPS of PCN.^[^ ^24^ ^]^ By contrast, PCN‐Rh‐0.5 shows a asymmetric peak, in which four fitted peaks are attributed to oxygen‐bridges (Rh─O─C) between triazine skeleton and Rh at 530.5 eV, hydroxyl groups (Rh─OH) on the Rh‐SAAs at 531.5 eV, hydroxyl groups (C─OH) on the PCN skeleton at 532.2 eV, and H_2_O from protonation of hydroxyl groups (Rh─OH_2_) at 532.9 eV, respectively.^[^ ^25^ ^]^ It suggests that the hydroxylated Rh‐SAAs are endowed with strong protonation ability to greatly improve hydrophilicity, which is also certified by their obviously reduced water contact angle relative to PCN (Figure S10, Supporting Information), thus promoting the PHE reaction.

To further investigate the electronic and geometric structure of Rh‐SAAs on the surface of PCN matrix, the XAFS is performed on PCN‐Rh‐0.5. X‐ray absorption near‐edge structure (XANES) is applied to identify Rh site structure based on the high sensitivity to the 3D arrangement of atoms around photo‐absorber. Figure 2d shows that the Rh *K*‐edge XANES of PCN‐Rh‐0.5 presents a profile similar to Rh_2_O_3_ reference, yet the pre‐edge peak intensity of PCN‐Rh‐0.5 is slightly increased in comparison with Rh_2_O_3_, suggesting the distorted octahedral coordination of the Rh atom because of the direct proportionality of coordination deviation of the photo‐absorber to centrosymmetry.^[^ ^26^ ^]^ The absorption edge position of Rh (half the height of the edge step) is almost identical to that of Rh_2_O_3_, demonstrating the dominant existence of Rh^3+^ in PCN‐Rh‐0.5.^[^ ^26^, ^27^ ^]^ Further local structural information of Rh atom is analyzed by Fourier transformations‐extended X‐ray absorption fine structure (FT‐EXAFS) in Figure 2e. PCN‐Rh‐0.5 depicts a major peak at ≈1.50 Å, which is very close to that of the RhO_6_ octahedra in Rh_2_O_3_ (∼1.53 Å), suggesting that the Rh─O bonding states are formed on the surface of PCN matrix through combining with the results obtained from FT‐IR spectra and XPS. Furthermore, the wavelet transform for EXAFS is carried out to more explicitly discriminate the coordination environment of Rh atom.^[^ ^26^ ^]^ As depicted in Figure 2f, markedly differing from Rh foil with an intensity maximum at ≈8.5 Å^−1^, PCN‐Rh‐0.5 has only one intensity maximum at ≈4.65 Å^−1^ that is very similar to that of Rh_2_O_3_ (≈4.85 Å^−1^), demonstrating the existence of single Rh atom centers without other crystal phases derived from them and also confirming the origin of the peak at ≈1.50 Å from Rh─O bonding states. Following, least‐squares curve fitting for EXAFS is carried out on the Rh moiety to extract quantitative coordination configuration (Figures S11–S16 and Table S3, Supporting Information). The average Rh─O bond length in PCN‐Rh‐0.5 is equal to that of Rh_2_O_3_ reference (2.02 Å) and the coordination numbers of Rh with O atoms in the first coordination sphere (5.7) are close to that of Rh_2_O_3_ reference (6), indicating an octahedral configuration of the Rh─O bonds. The further DFT calculations (Figure S17, Supporting Information) confirm the hydroxylated Rh‐SAAs with octahedral configuration (inset of Figure 2e) are assembled on the surface of PCN matrix by dual oxygen‐bridges. This unique aerial configuration with rigid dual oxygen‐bridges can not only fully expose the Rh atom sites but also effectively conquer the lattice distortion caused by nitrogen defects, thereby achieving efficient, stable, and long‐term PHE reactions.

**Figure 3:**
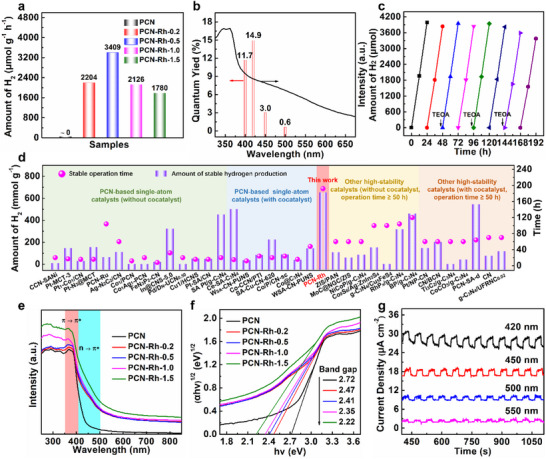
a) Average PHE rate of the samples. b) AQY at different wavelengths and c) cyclic PHE curves of PCN‐Rh‐0.5. d) Stability comparison of PCN‐Rh‐0.5 with reported PCN‐based single‐atom catalysts and other high‐stability catalysts with operation time ≥50 h under the same reaction conditions. e) UV–vis DRS and f) Tauc plots of all samples. g) Transient photocurrent responses of PCN‐Rh‐0.5 at different wavelengths.

The PHE performance of the samples is evaluated under visible light (*λ* ≥420 nm) without additional catalysts or sensitizers. From kinetics curves (Figure S18, Supporting Information) and corresponding average rates depicted in **Figure** **3**a, the average PHE rate over the optimal PCN‐Rh‐0.5 reaches to 3409 µmol g^−1^ h^−1^ and is 32.5 times that of the PCN/Pt benchmark (Figure S19, Supporting Information). The apparent quantum yield (AQY) of PHE over PCN‐Rh‐0.5 is 14.9% at 420 nm (Figure 3b). Astonishingly, PCN‐Rh‐0.5 achieves a longer operation time (192 h) with a higher amount of hydrogen production per unit mass (654.5 mmol g^−1^) than the PCN‐based single‐atom catalysts reported and other high‐stability catalysts with operation time ≥50 h under the same conditions (Figure 3c,d; Tables S4 and S5, Supporting Information). The structural stability is further evidenced by almost unchanged XRD patterns (Figure S20, Supporting Information), FT‐IR spectra (Figure S21, Supporting Information), and TEM image (Figure S22, Supporting Information) of PCN‐Rh‐0.5 after cyclic reactions.

Figure 3e shows the UV–vis diffuse reflectance spectra (DRS) of samples. Compared to PCN, all PCN‐Rh examples depict the intensified *π*−*π* ^*^ transition absorption band of the conjugated system ≈350−390 nm and the significantly redshift absorption edges with increasing Rh content.^[^ ^28^, ^29^, ^30^ ^]^ Meanwhile, the forbidden *n*−*π* ^*^ transition involving the lone pair electrons of N atoms in the s‐triazine rings is allowed and enhanced ≈410−470 nm, which is due to the slight symmetry deviation resulting from the surface modification of hydroxylated Rh‐SAAs on the surface of PCN matrix.^[^ ^28^, ^29^, ^30^ ^]^ Noticeably, all PCN‐Rh samples show an obvious Urbach tail absorption in the visible light region above 500 nm, attributed to the electronic localized state in the trap level within the bandgap caused by modification of Rh‐SAAs on the surface of PCN matrix, which is also reflected by the uneven regions in front of valence band (VB) edge of PCN‐Rh‐0.5 compared with PCN (Figures S23 and S24, Supporting Information).^[^ ^31^, ^32^ ^]^ Moreover, based on Tauc plots drawn from UV–vis DRS (Figure 3f),^[^ ^33^ ^]^ the calculated bandgap of samples is gradually narrowed from 2.72 to 2.22 eV with the increase of Rh content. The above improved light harvesting and utilization ability of PCN‐Rh samples is further confirmed by the produced photocurrent responses at several fixed wavelengths originated from the *n*−*π* ^*^ electronic transition and improved localized electronic states in the bandgap (Figure 3g). The XPS VB spectra (Figures S23 and S24, Supporting Information) and Mott–Schottky plots (Figures S25 and S26, Supporting Information) demonstrate the unchanged VB position and the down‐shift CB position closer to Fermi level for PCN‐Rh‐0.5 relative to PCN, suggesting that the energy band structure is adjusted effectively via the assembly of hydroxylated Rh‐SAAs on the surface of PCN matrix to promote PHE reaction (Figure S27, Supporting Information).^[^ ^19^ ^]^

**Figure 4:**
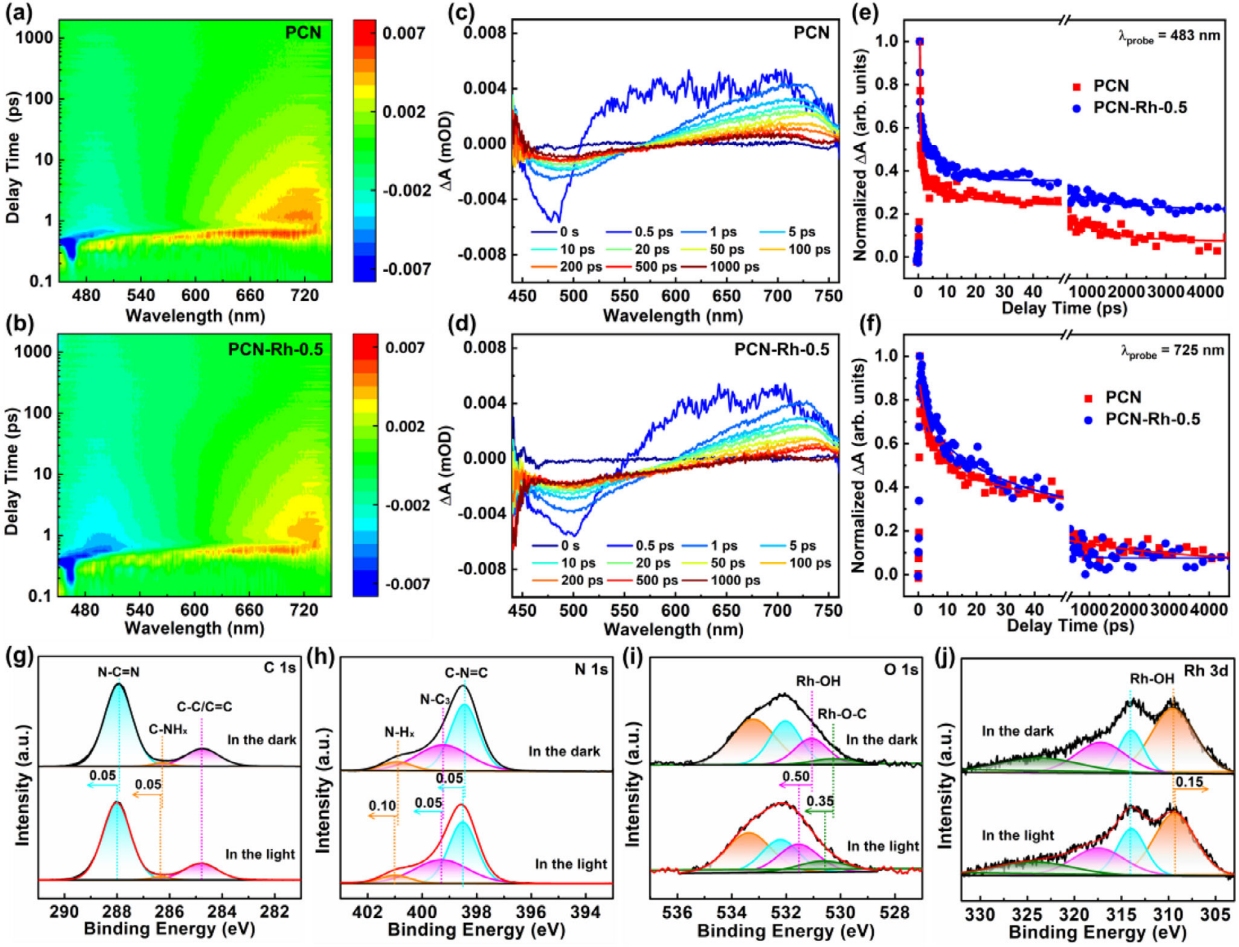
a,b) Pseudocolor plots, c,d) fs‐TA spectra with different delay times, and e,f) kinetics decay curves at 483 and 725 nm of PCN and PCN‐Rh‐0.5 under a 400 nm pump. g–j) In situ XPS of PCN‐Rh‐0.5 in the dark and light.

To essentially gain insight into the effect of hydroxylated Rh‐SAAs on charge transfer behavior in the PHE reaction, the fs‐TA spectra are first employed to monitor the population of trapped states and the decay kinetics of charge carriers for the samples. **Figure** **4**a–d show the pseudocolor plots and kinetic decay curves of the samples. For PCN, the negative broad absorption from 450 to 570 nm is derived from ground state bleach (GSB) signal related to photogenerated holes on VB, while the positive characteristic broad peaks at long wavelength region between 450 and 730 nm are attributed to the excited state absorption (ESA) originated from photogenerated electrons on CB.^[^ ^34^, ^35^ ^]^ By contrast, PCN‐Rh‐0.5 shows enhanced GSB and weakened ESA signals, suggesting the injection of photogenerated electrons from PCN skeleton into hydroxylated Rh‐SAAs and thus improving carrier separation efficiency,^[^ ^36^ ^]^ To deeply figure out the kinetic behaviors of charge carriers, the decay curves of fs‐TAS at 483 nm are fitted by using a three‐exponential mode. As shown in Figure 4e and Table S6 (Supporting Information), more than 90% of ultrafast component (τ_1_) of two samples is attributed to the recombination or surface state capture of free photogenerated electrons, in which the slight lifetime extension (0.19 ps) detected on PCN‐Rh‐0.5 compared to PCN (0.17 ps) may be attributed to the surface optimization originated from modification of hydroxylated Rh‐SAAs.^[^ ^35^ ^]^ Less than 3.1% of the slow component (τ_3_) of two samples has an ultra‐long lifetime of ≈1000 ps, which arises from the unfavorable exciton recombination state.^[^ ^36^ ^]^ Besides, the proportion of fast component (τ_2_) for PCN‐Rh‐0.5 is increased, but the lifetime (6.76 ps) is shortened compared with PCN (10.66 ps), which suggests that the additional trap level is produced owing to the surface modification of hydroxylated Rh‐SAAs, thereby improving the separation efficiency of charge carriers.^[^ ^37^ ^]^ Moreover, the decay curves of fs‐TAS at 725 nm are further fitted by using the three‐exponential mode and depicted in Figure 4f and Table S7 (Supporting Information). Noticeably, PCN shows an ultra‐long lifetime of over 3200 ps (τ_3_), which indicates that a large number of photogenerated electrons may be in an exciton recombination state and thereby are difficult to participate in the photocatalytic reaction. However, the lifetime of τ_3_ for PCN‐Rh‐0.5 is decreased to 262 ps, suggesting its effective exciton fission via the additional trap level produced by surface modification of hydroxylated Rh‐SAAs, thus dramatically promotes the spatial separation of photogenerated charges and extend their diffusion distance and lifetime.^[^ ^38^, ^39^ ^]^ Moreover, compared to PCN, PCN‐Rh‐0.5 displays the reduced PL intensity (Figure S28, Supporting Information), prolonged average lifetime extracted from TR‐PL decay curves (Figure S29 and Table S8, Supporting Information), and enhanced photocurrent density (Figure S30, Supporting Information), suggesting that the separation of photogenerated electron–hole pairs is obviously promoted because of the directed transfer of photogenerated electrons from PCN skeleton to hydroxylated Rh‐SAAs via the formed trap level.^[^ ^40^ ^]^ Further measurements of electrochemical properties show that PCN‐Rh‐0.5 has smaller Nyquist curve radius and charge transfer resistance, lower overpotential and Tafel slop, and larger double‐layer capacitance (C_dl_) and electrochemical active surface area (ECSA) than PCN (Figures S31–S36, Supporting Information), which demonstrates its significantly decreased interface charge transfer resistance, accelerated hydrogen evolution kinetic, and more abundant active sites, thus being favorable for PHE reaction.^[^ ^40^ ^]^ To further prove the directed transfer of photogenerated electrons, in situ XPS was performed on PCN‐Rh‐0.5. As shown in Figure 4g–j, compared to XPS in the dark, the peaks of C 1s, N 1s, and O 1s states move toward high binding energy while those of Rh 3d state shift toward the opposite direction in the light, confirming the injection of photogenerated electrons from PCN skeleton into hydroxylated Rh‐SAAs.^[^ ^41^ ^]^

**Figure 5:**
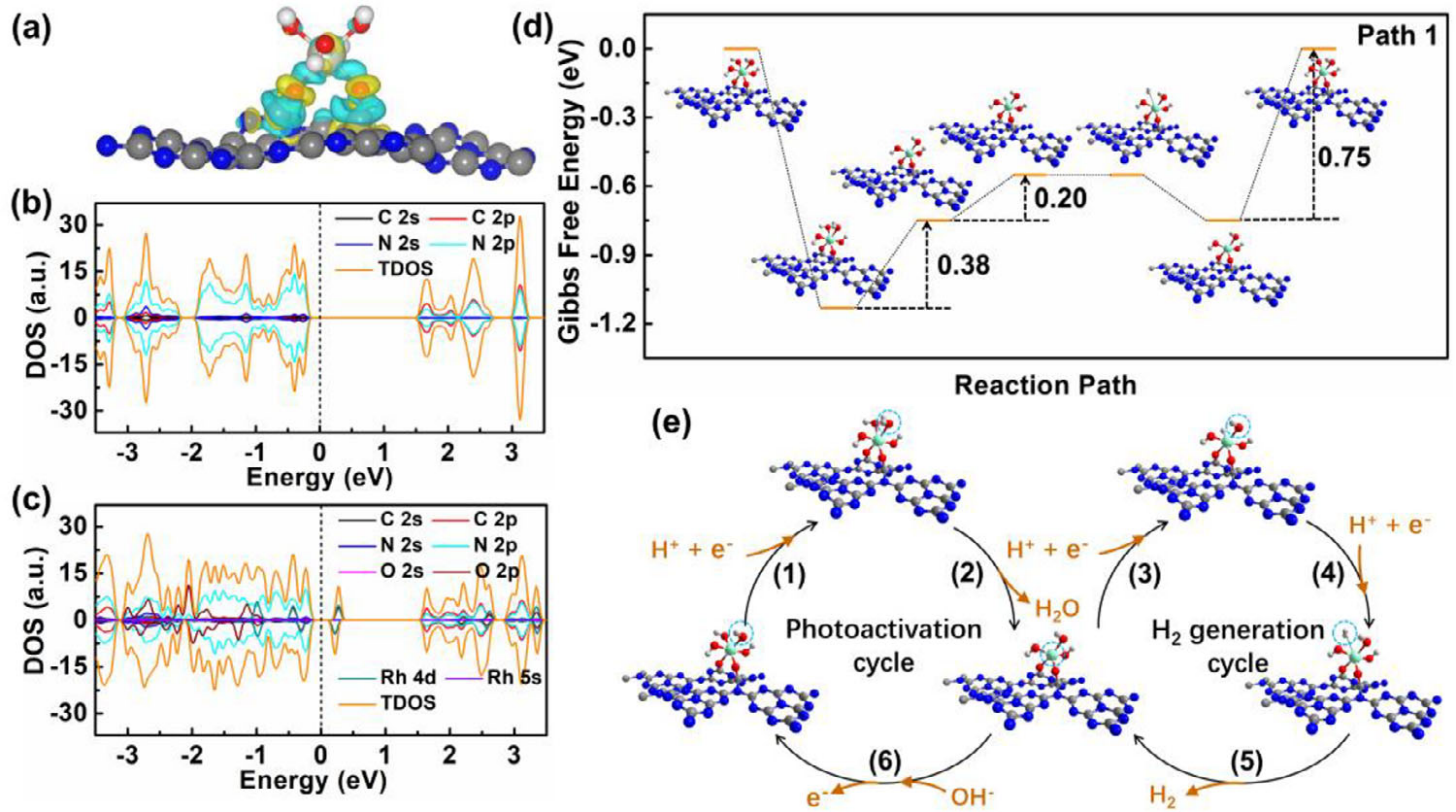
a) Charge density difference diagram (yellow and cyan represent the accumulation and loss of electrons, respectively), b,c) DOS of PCN and PCN‐Rh, d) free energy diagrams, and e) proposed PHE mechanism on PCN‐Rh.

To uncover the inner driving force of charge transfer, the interfacial charge redistribution between the hydroxylated Rh‐SAAs and PCN matrix is investigated based on DFT calculations. As the charge density difference diagram shown in **Figure** **5**a, the spontaneous electron redistribution results in an excess of electrons on the hydroxylated Rh‐SAAs, implying that it has a strong electron‐withdrawing ability to promote photogenerated charge separation.^[^ ^42^ ^]^ Further Bader charge analysis (Figure S37 and Table S9, Supporting Information) indicates that the hydroxylated Rh‐SAAs with −1.71 e^−^ charge can achieve hydrogen evolution through the preferential protonation. Additionally, compared the calculated band structure (Figures S38 and S39, Supporting Information) and density of states (DOS) (Figure 5b,c) of PCN‐Rh with those of PCN, a new energy level appears above Fermi level through the hybridization of Rh 4d and O 2p orbitals, which as trap level captures electrons in the exciton recombination state to drive exciton fission and then plays an electron pump role to promote the directed electron transfer to the hydroxylated Rh‐SAAs.^[^ ^36^ ^]^ Furthermore, based on DFT calculations, four reaction paths proposed are likely to occur on the hydroxylated Rh‐SAAs (Figure 5d,e; Figures S40–S42, Supporting Information). The free energy profiles show that the hydroxyl group combining with a proton tends to be a spontaneous process in the first step, indicating the strong protonation of hydroxylated Rh‐SAAs on the surface of PCN matrix. Noticeably, the optimal Path 1 follows by a dual‐cycle process in the PHE reaction, including a photoactivation cycle and a hydrogen generation cycle. It suggests that the continuous hydrogen evolution can be achieved by overcoming an energy barrier as low as 0.20 eV once PCN‐Rh is activated in the light. All in all, the PHE activity is effectively improved through a dual‐cycle reaction path triggered by the protonation of hydroxylated Rh‐SAAs on the surface of PCN matrix.

## 3 Conclusion

In summary, a PCN‐Rh single‐atom catalyst is fabricated employing LEP strategy, which achieves long‐term, efficient, and stable PHE reaction under visible light (λ ≥420 nm) without additional cocatalyst owing to its unique hydroxylated Rh‐SAAs configuration. We confirm the atomic‐level assemble structure with hydroxylated Rh‐SAAs connected by dual oxygen‐bridge on the surface of PCN matrix on the basis of XAFS and XPS analyses combined with DFT calculations. The fs‐TA spectra, TR‐PL decay curves, in situ XPS, and some other advanced characterization techniques evidence that the excellent PHE performance originates from the improved separation efficiency and lifetime of photogenerated charges owing to the trap level induced electron pump effect derived from hydroxylated Rh‐SAAs. Moreover, DFT calculations indicate that the strong protonation of hydroxylated Rh‐SAAs triggers the PHE reaction to follow a dual‐cycle reaction path. The optimal PCN‐Rh‐0.5 reaches 32.5 times the average PHE rate of the PCN/Pt benchmark. Meanwhile, PCN‐Rh‐0.5 shows a longer operation time of 192 h with the higher amount of hydrogen production of 654.5 mmol g^−1^ during the stability test than the reported PCN‐based single‐atom catalysts and other high‐stability catalysts with operation time ≥50 h. These findings highlight the significance of atomic configuration design of single‐atom catalysts for photocatalytic activity and stability, and endow a fresh understanding of PHE mechanism. This work not only provides an effective strategy for the development of efficient and high‐stability photocatalysts, but also offers a successful paradigm for the structure engineering of single‐atom/2D matrix extensible to other systems.

## Conflict of Interest

The authors declare no conflict of interest.

## Supporting information



Supporting Information

## Data Availability

The data that support the findings of this study are available from the corresponding author upon reasonable request.
