# Supplementary material for: Hydroxylated Rh Single‐Atom Antennas Assembled on Carbon Nitride Toward Stable Photocatalytic Hydrogen Evolution
Source: Adv Sci (Weinh). 2025 Dec 16;13(6):e18847. doi: 10.1002/advs.202518847 (PMC12866703; doi:10.1002/advs.202518847)
Supplement: Supplementary file 1 — Supporting Information [file ADVS-13-e18847-s001.docx]

**Supporting Information**

**Hydroxylated Rh Single-Atom Antennas Assembled on Carbon Nitride towards Stable Photocatalytic Hydrogen Evolution**

Chunmei Li, Pingfan Zhang, Ming Zheng, Shasha Cheng, Baodong Mao, Guangbo Che*, Song Wang*, Weidong Shi*, and Hongjun Dong*

*Corresponding author: guangboche@bcnu.edu.cn, wangsong1984@126.com, swd1978@ujs.edu.cn, hjdong@ujs.edu.cn

**Experimental section**

**1. Chemicals**

Urea (AR, ≧99%), Rhodium (III) acetylacetonate (AR, 97%), H_2_PtCl_6_·H_2_O (AR, ≧99%), triethanolamine (AR, ≧78%), ethanol (AR, ≧99.7%) and deionized water were directly used without further treatment.

**2. Synthesis of photocatalysts**

Acetylacetone rhodium alcohol solution (3.5 mL) was slowly dropped into an aqueous solution (30 mL) containing urea (20 g), and ultrasonic dispersion for 20 min. Heat in a sealed oil bath at 90 ℃ and stir for 4.5 h before drying. The obtained solid was dried overnight in a vacuum drying oven and then calcined for 4 h at 550 ℃ with a heating rate 2.5 ℃ min^-1^. The crude product of PCN-Rh photocatalyst was obtained. The crude product was further pickled in a nitric acid solution with a volume fraction of 1.5% for 5 h, washed alternately with water and ethanol for several times, and then dried overnight. After grinding evenly, the PCN-Rh photocatalyst was finally obtained, denoted as PCN-Rh-X (X = 0.2, 0.5, 1, 1.5) based on corresponding amount of acetylacetone rhodium (7, 18, 35, 53 mg). The preparation method of pure PCN is the same as that of PCN-Rh, without adding acetylacetone rhodium.

**3. Characterizations**

The crystallinity of samples was investigated by X-ray diffraction (XRD) on SHIMADZU XRD-6100 diffractometer (10-80°). The Fourier transform infrared (FT-IR) spectra were measured on a Nicolet IS 50 spectrometer with KBr as the reference dispersion medium. The transmission electron microscope (TEM) images were obtained by JEOL JEM-2011 (HR) and FEI Talos F200x G2 instruments. The spherical aberration-corrected transmission electron microscope (SAC-TEM) images were collected on FEI Theims Z instrument. X-ray photoelectron spectra (XPS) were detected by ThermoFisher-Nexsa. The electron paramagnetic resonance spectra (EPR) were obtained by Bruker A300-10/12 instrument. The UV-vis diffuse reflectance spectra (DRS) were recorded by UV-vis spectrophotometer (UV-3600). The Brunauer-Emmett-Teller (BET) specific surface area and the pore size distribution were measured using a BEL SORP instrument. Photoluminescence (PL) spectroscopy and time-resolved photoluminescence (TR-PL) decay curves were detected on a FluoroMax-4C-Tcspc luminescence spectrometer. X-ray absorption spectra (XAS) were collected on National Synchrotron Light Source of Brookhaven National Laboratory.

**4. Photocatalytic hydrogen evolution experiments**

Photocatalyst (50 mg) was evenly dispersed in aqueous solution containing triethanolamine (TEOA, 10 Vol%, 100 mL) in a quartz reactor and the air was exhausted by nitrogen for 30 min. The visible light was provided by a 300 W xenon lamp (PLS-SXE300UV) equipped with a 420 nm cut-off filter. During the reaction, circulating water (10 ℃) was used to maintain the temperature of reaction system and hydrogen production was determined by gas chromatography (GC-2030). The apparent quantum yield (AQY) was measured under the monochromatic LED lamps (400, 420, 450, and 500 nm).

**5. Photoelectrochemical and electrochemical measurements**

The photoelectrochemical and electrochemical properties of the photocatalysts were measured by the electrochemical workstation VersaSTAT 3A with a standard three-electrode system. 300 W xenon lamp with a 420 nm cut-off filter and the monochromatic LED lamps (420, 450, 500, and 550 nm) were used as the light source. The electrolyte was Na_2_SO_4_ solution (0.5 M). The reference electrode and counter electrode were saturated Ag/AgCl reference electrode and platinum plate electrode respectively. Photocatalyst (50 mg), oleic acid (0.03 mL), and PVP (0.01 g) were added into ethanol (3 mL) to form homogeneous suspension by ultrasonic dispersion for 6 h. The obtained suspension was rotated on ITO glass (2×2 cm) at 2000 r min^-1^ for 3 min, and thus the working electrode was obtained after drying.

**6. Theory calculations**

The spin-polarized density functional theories (DFT) were carried out by using the Vienna Ab initio Simulation Package (VASP). ^[1,2]^ The Perdew-Burke-Ernzerhof generalized-gradient approximation functional was used to describe the interaction between electrons. ^[3]^ The D3 correction of Grimme ^[4]^ was adopted to compensate for the lack of van der Waals interaction description in the GGA functional. The energy cutoff was set to 450 eV. The Monkhorst-Pack k-points grid was set to be 3×3×1 for RhOH/C_3_N_4_ during the calculations. The vacuum region was set to be 15Å in z direction to prevent the interaction between two adjacent surfaces. The energy convergence was set to 10^-5^ eV. Bader charge analysis ^[5,6]^ was used to observe the behavior of electronic charge transfer in atoms on the outer surface.

The reaction Gibbs free energy (ΔG) is defined as ΔG = ΔE + ΔΕ_ZPE_ - TΔS, where ΔE is the reaction energy, ΔΕ_ZPE_ is zero-point energies, T is the temperature (298.15 K), ΔS is the difference in entropy from vibrational frequency calculations. The zero point can also contain insignificant internal energy changes, which are uniformly corrected and processed by the VASPKIT ^[7]^.

The adsorption energy (E_ads_) is expressed as: E_ads_ =E _total_ – (E _adsorbate_ +E _surface_), where E _total_ is the total energy of the adsorbed surface, E _adsorbate_ is the energy of the adsorbed surface, and E _surface_ is the energy of the pure surface. By empirical definition, the negative value of E _ads_ represents the energy released or relatively stable adsorption.


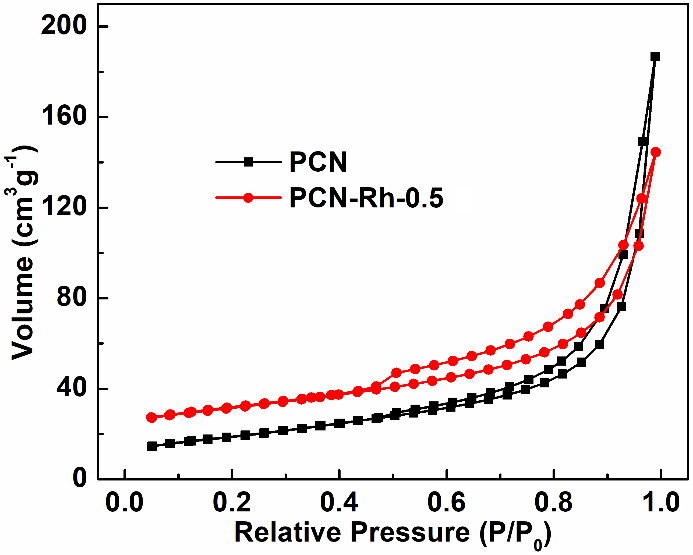


**Figure. S1** N_2_ adsorption-desorption isotherms of PCN and PCN-Rh-0.5.


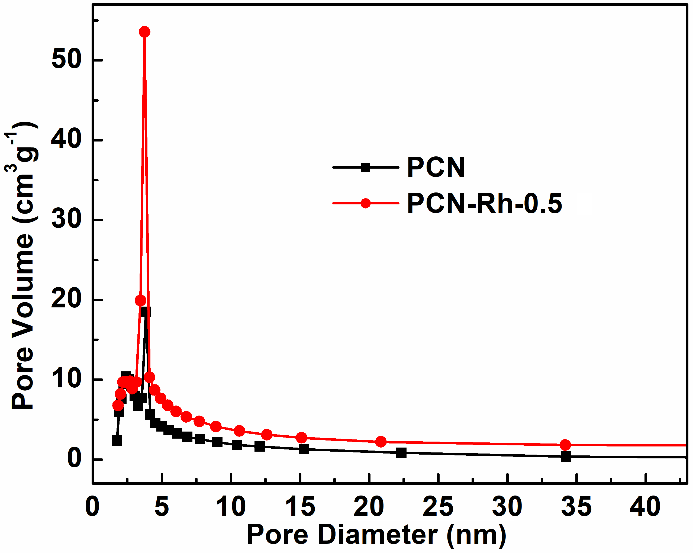


**Figure. S2** Pore size distributions of PCN and PCN-Rh-0.5.


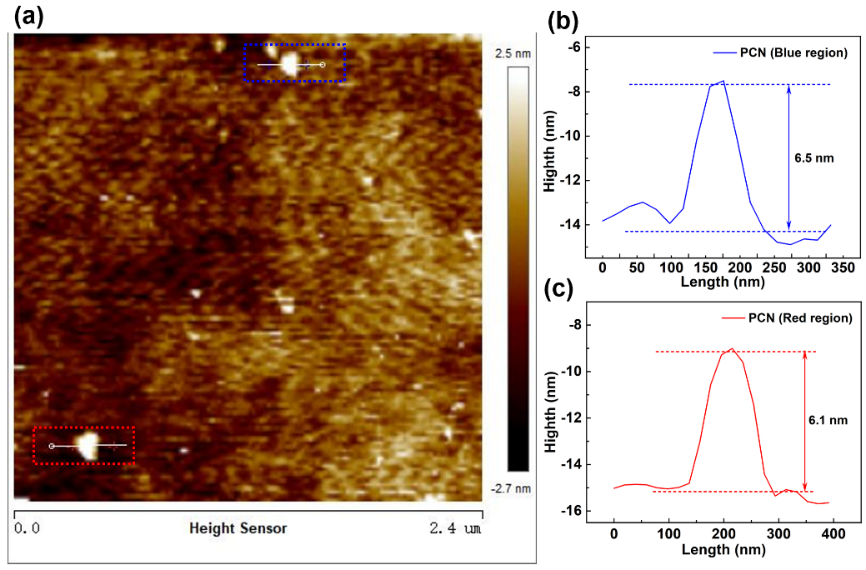


**Figure. S3** (a) AFM image and (b, c) thickness of marked nanosheet in AFM image of PCN.


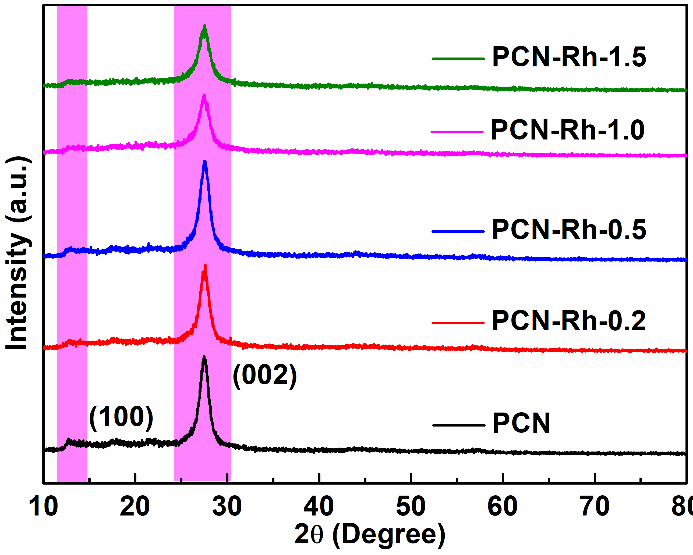


**Figure. S4** XRD of PCN and different PCN-Rh samples.


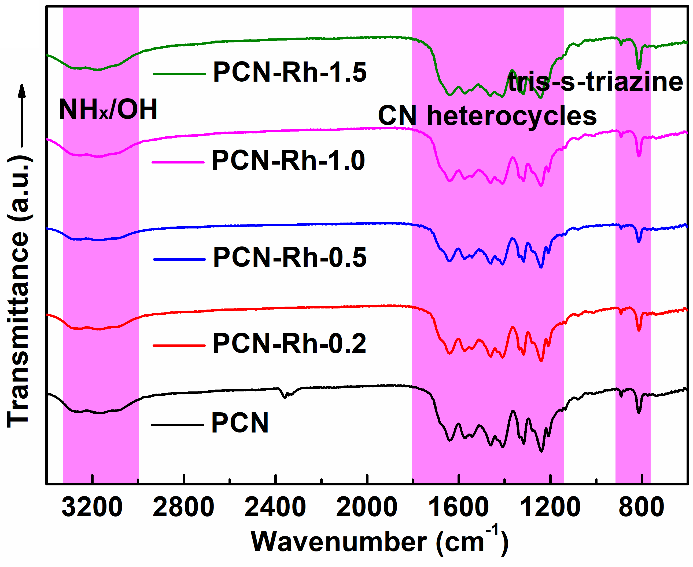


**Figure. S5** FT-IR spectra of PCN and different PCN-Rh samples at 3400-600 cm^-1^.


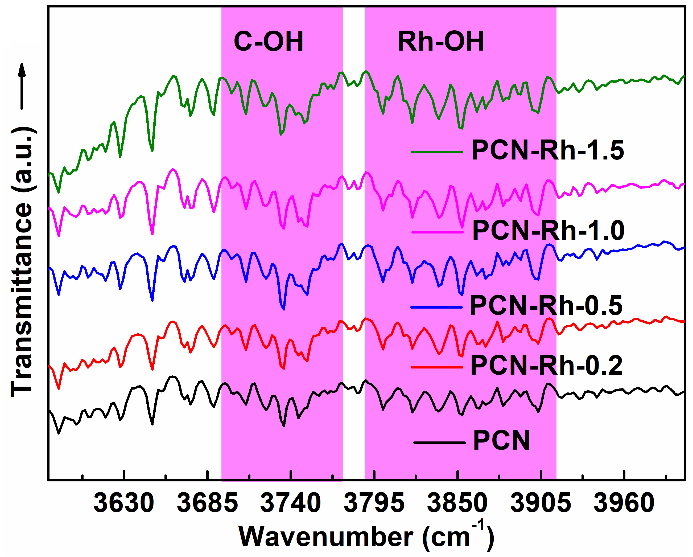


**Figure. S6** FT-IR spectra of PCN and different PCN-Rh at 4000-3580 cm^-1^.


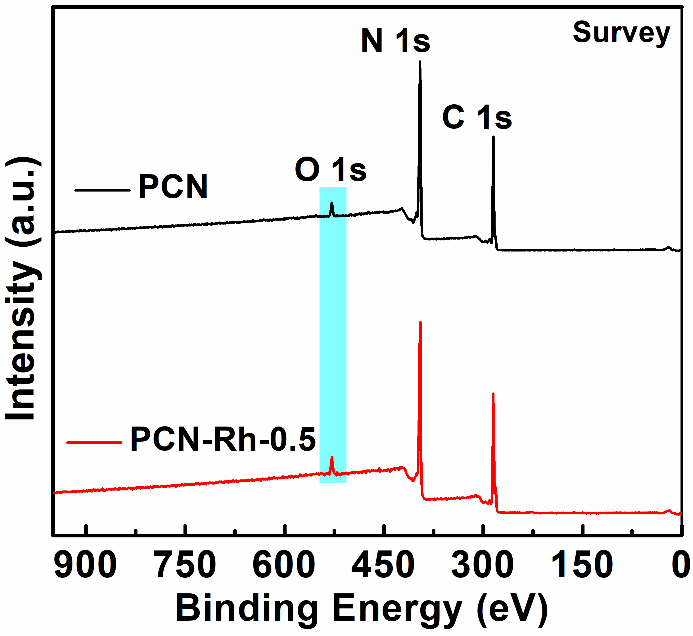


**Figure. S7** Survey XPS of PCN and PCN-Rh-0.5.


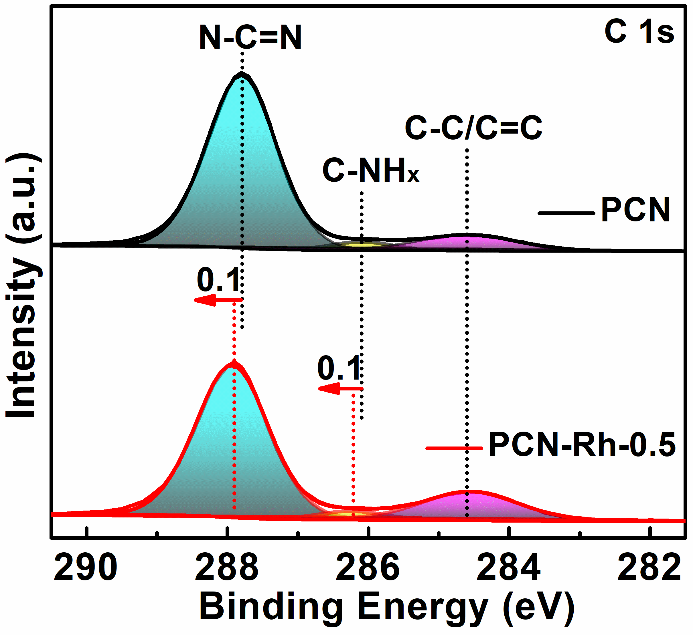


**Figure. S8** C 1s XPS of PCN and PCN-Rh-0.5.


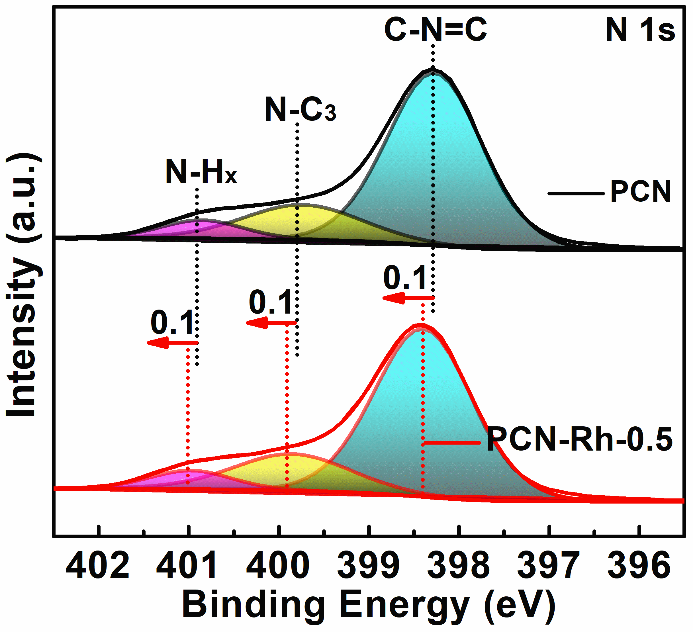


**Figure. S9** N 1s XPS of PCN and PCN-Rh-0.5.


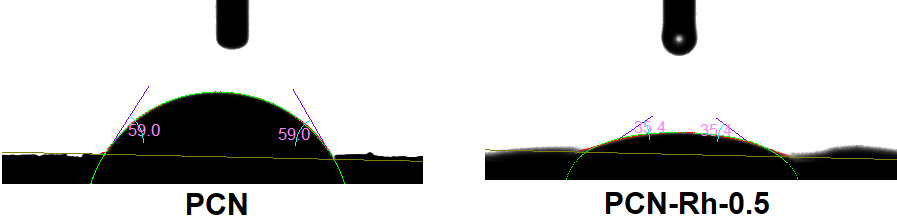


**Figure. S10** Water contact angle of PCN and PCN-Rh-0.5.


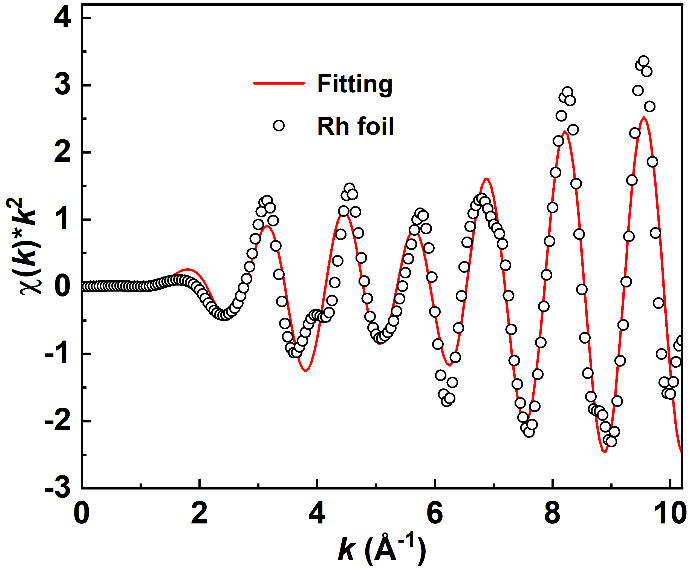


**Figure. S11** k space fitting curves for Rh foil.


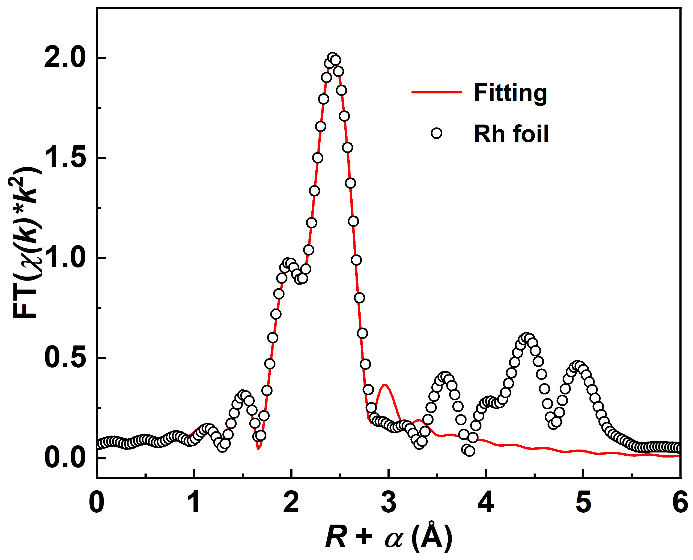


**Figure. S12** R space fitting curves for Rh foil.


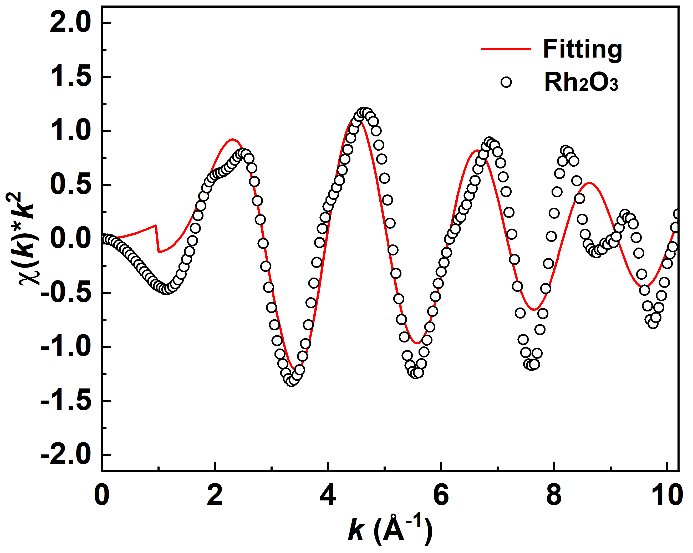


**Figure. S13** k space fitting curves for Rh_2_O_3_.


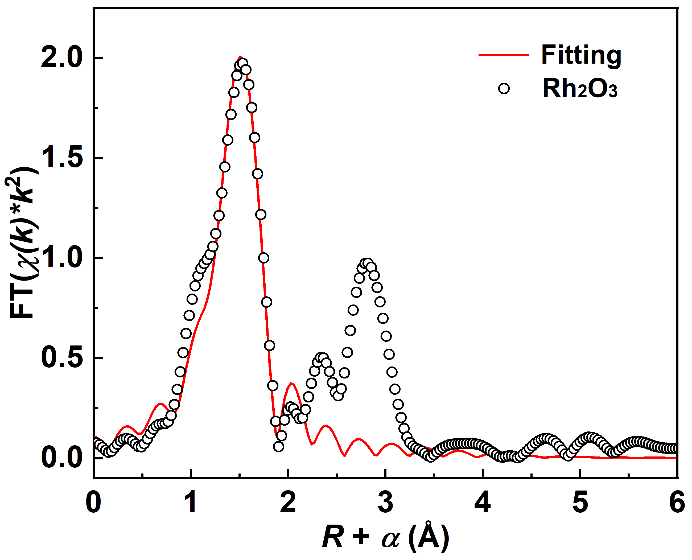


**Figure. S14** R space fitting curves for Rh_2_O_3_.


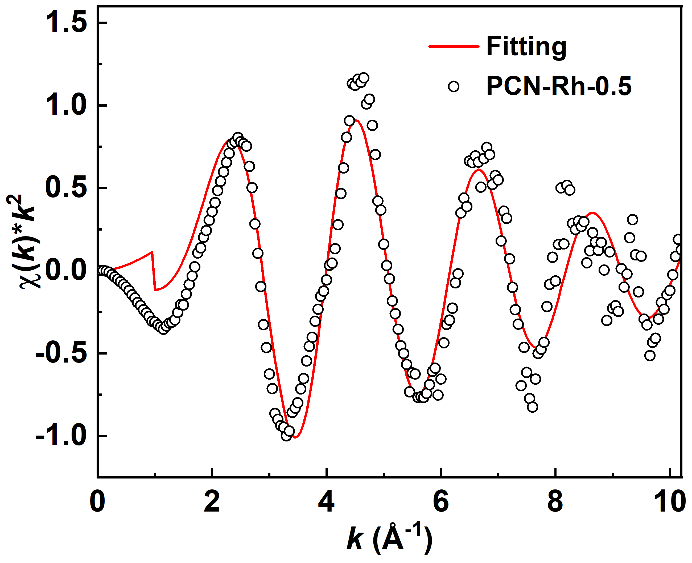


**Figure. S15** k space fitting curves for PCN-Rh-0.5.


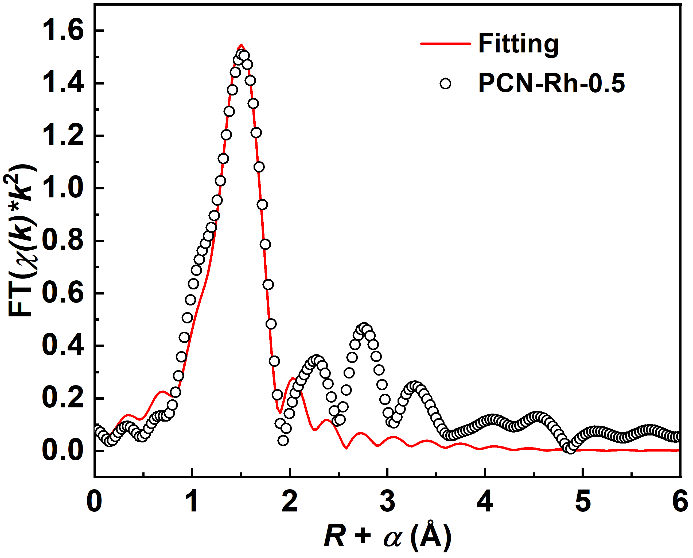


**Figure. S16** R space fitting curves for PCN-Rh-0.5.


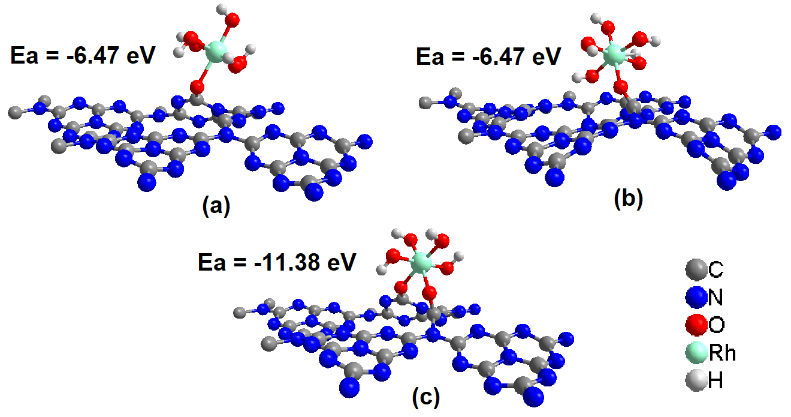


**Figure. S17** Possible structure models of PCN-Rh.


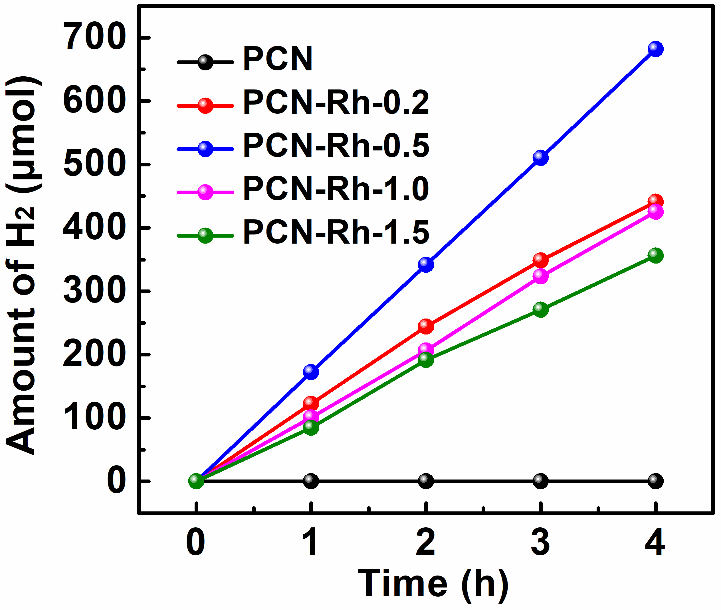


**Figure. S18** Kinetic curves over the different samples.


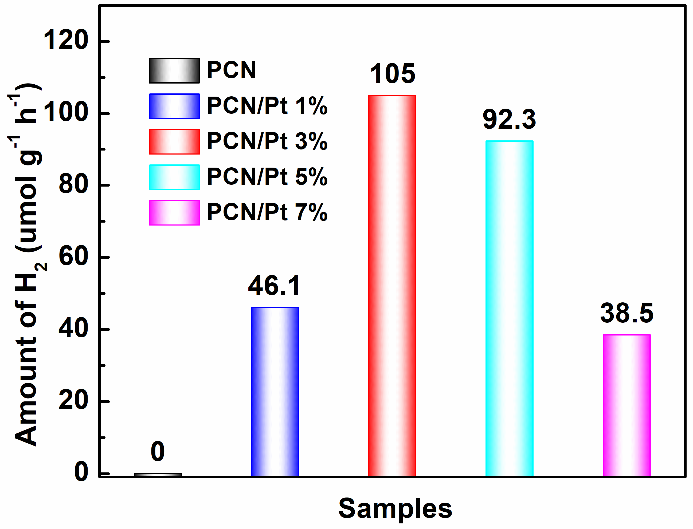


**Figure. S19** Average PHE rate over the different samples.


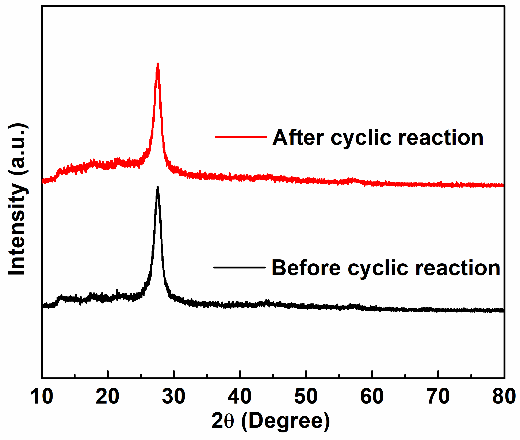


**Figure. S20** XRD of PCN-Rh-0.5 before and after cyclic reaction.


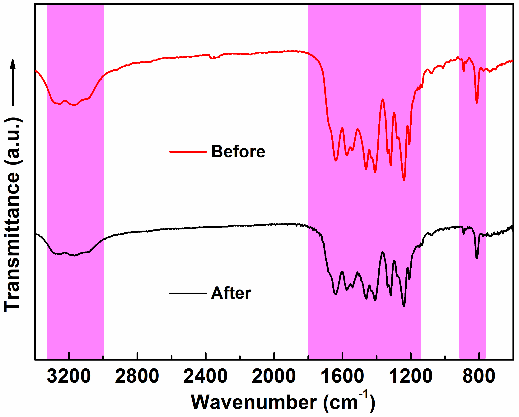


**Figure. S21** FT-IR spectra of PCN-Rh-0.5 before and after cyclic reaction.


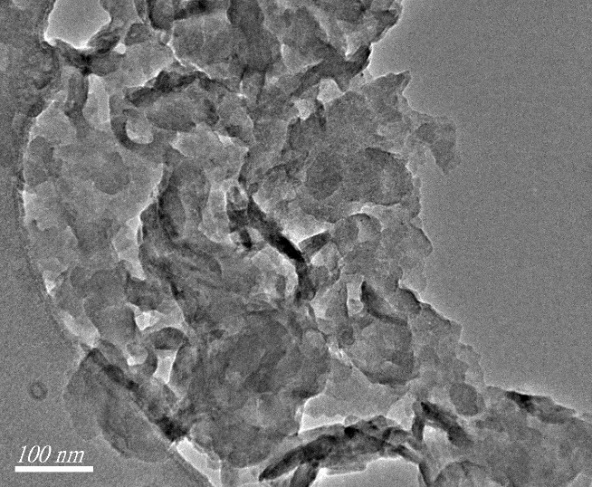


**Figure. S22** TEM image of PCN-Rh-0.5 after cycle reaction.


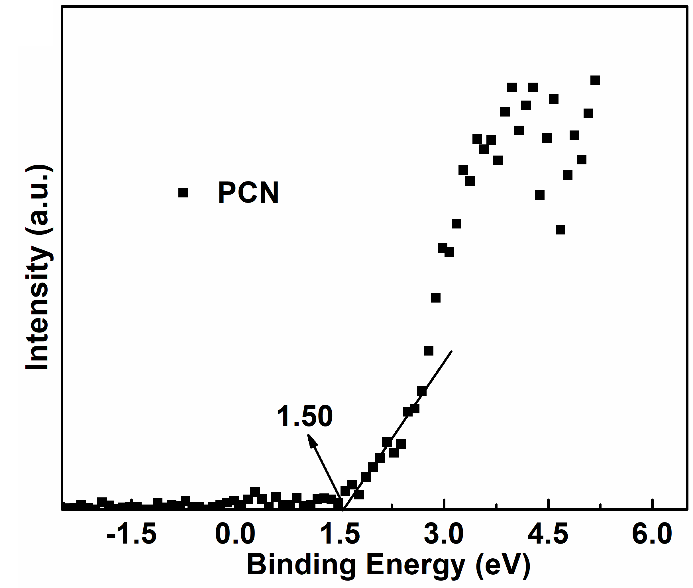


**Figure. S23** Valence band spectrum of PCN.


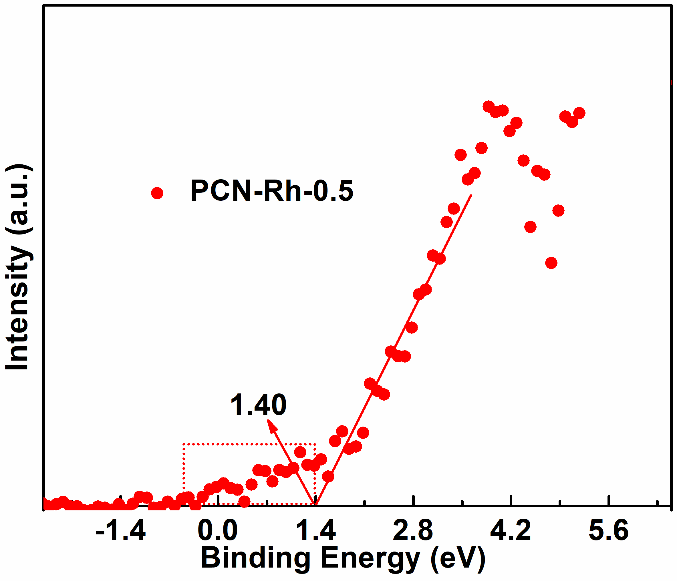


**Figure. S24** Valence band spectrum of PCN-Rh-0.5.


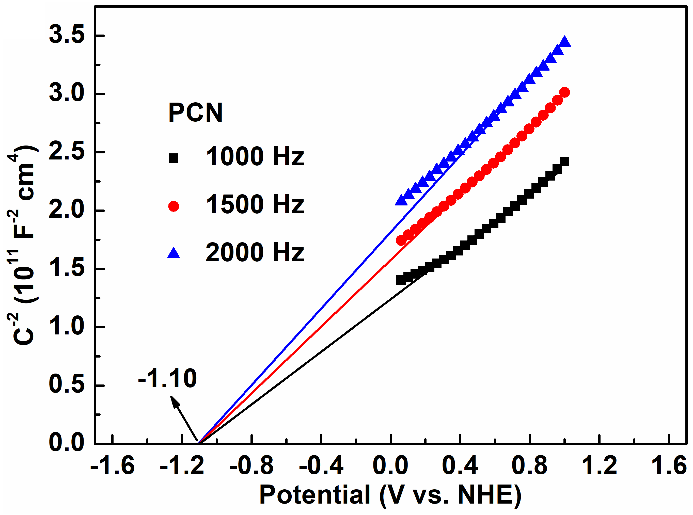


**Figure. S25** Mott-Schottky plots of PCN.


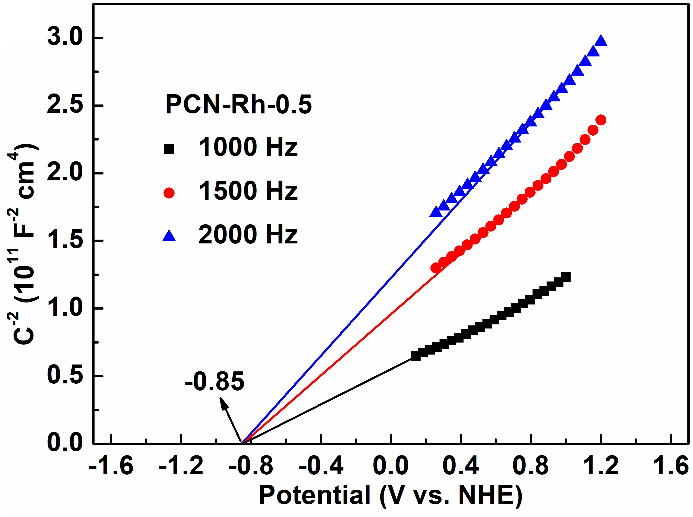


**Figure. S26** Mott-Schottky plots of PCN-Rh-0.5.


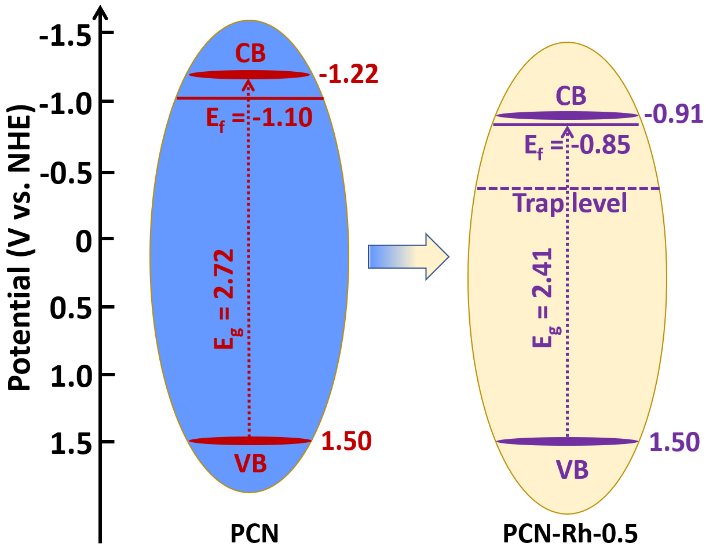


**Figure. S27** Energy band structure schematic of PCN and PCN-Rh-0.5.


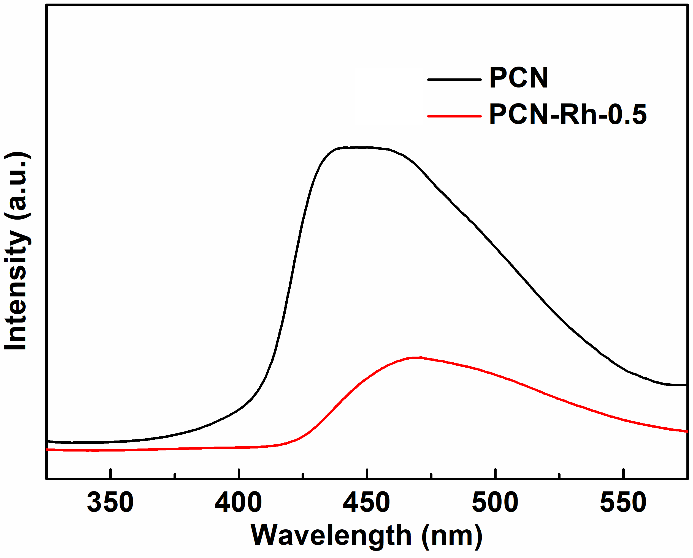


**Figure. S28** PL spectra of PCN and PCN-Rh-0.5.


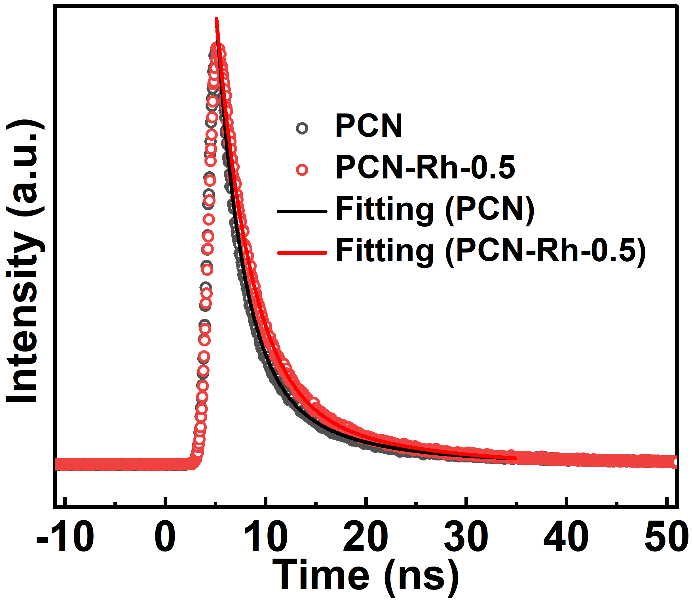


**Figure. S29** TR-PL decay curves of PCN and PCN-Rh-0.5.


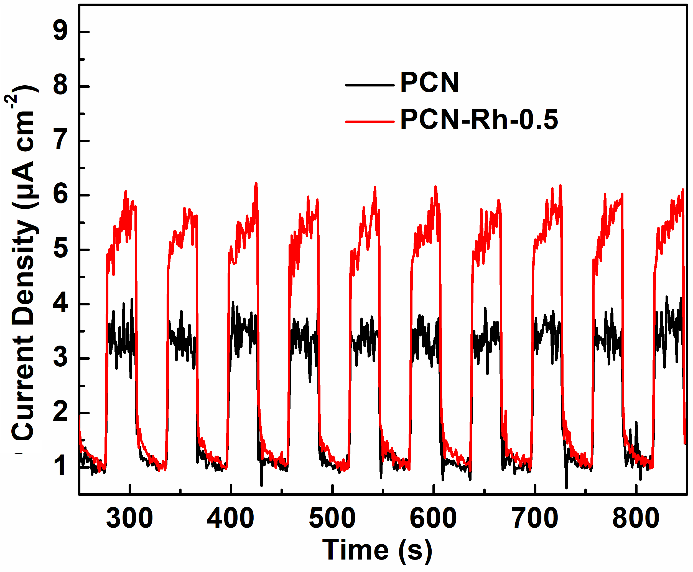


**Figure. S30** Transient photocurrent response of PCN and PCN-Rh-0.5.


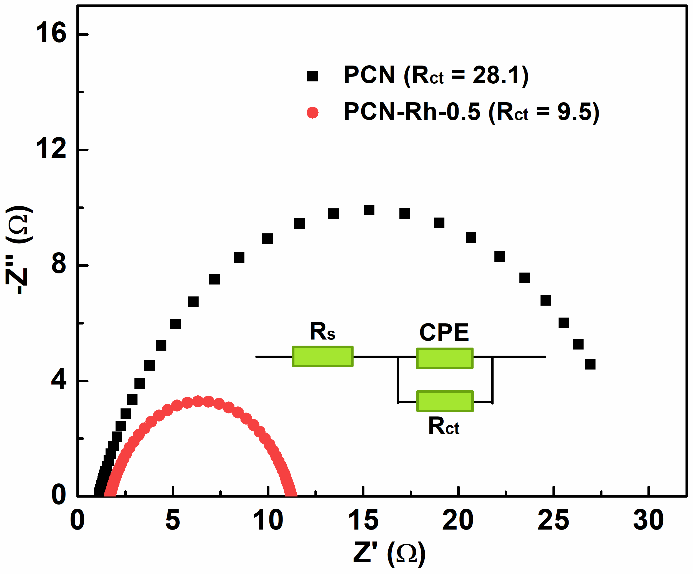


**Figure. S31** EIS Nyquist plots of PCN and PCN-Rh-0.5.


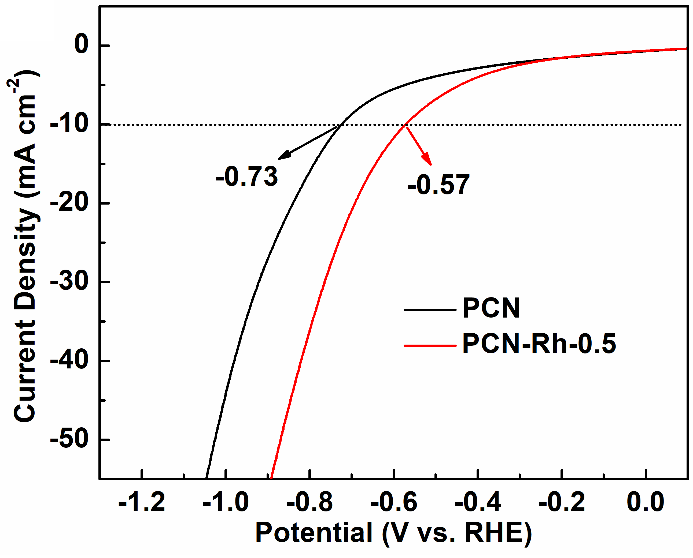


**Figure. S32** LSV curves of PCN and PCN-Rh-0.5.


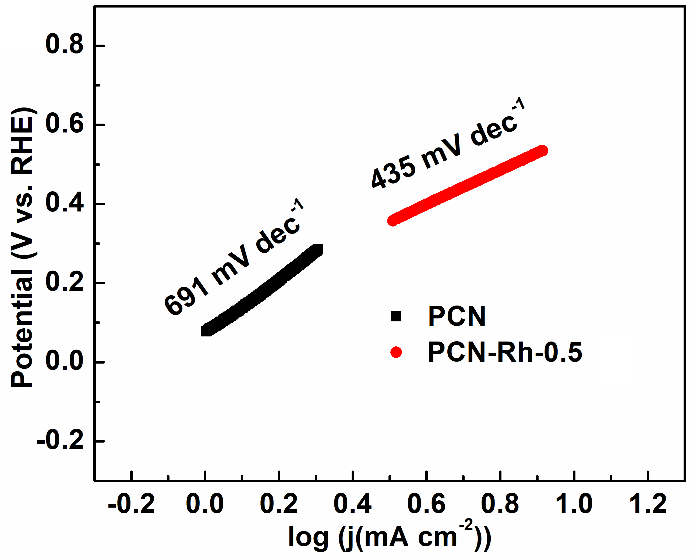


**Figure. S33** Tafel plots of PCN and PCN-Rh-0.5.


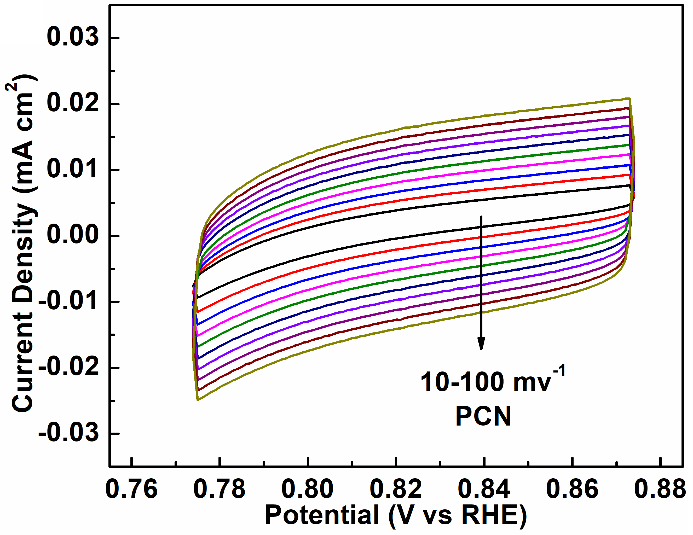


**Figure. S34** CV curves of PCN.


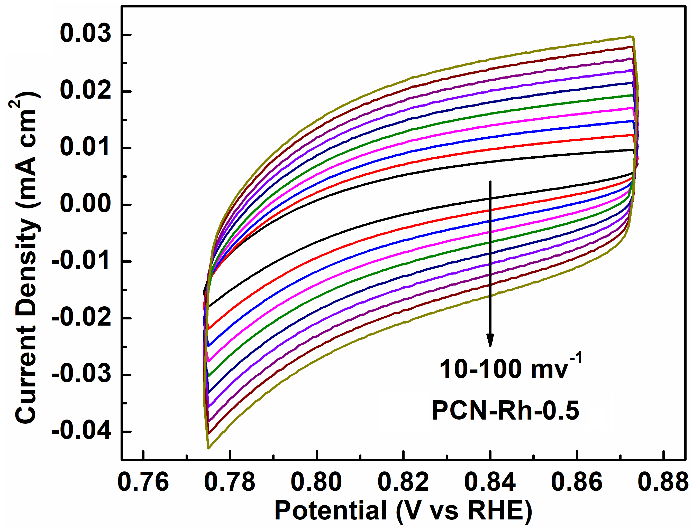


**Figure. S35** CV curves of PCN-Rh-0.5.


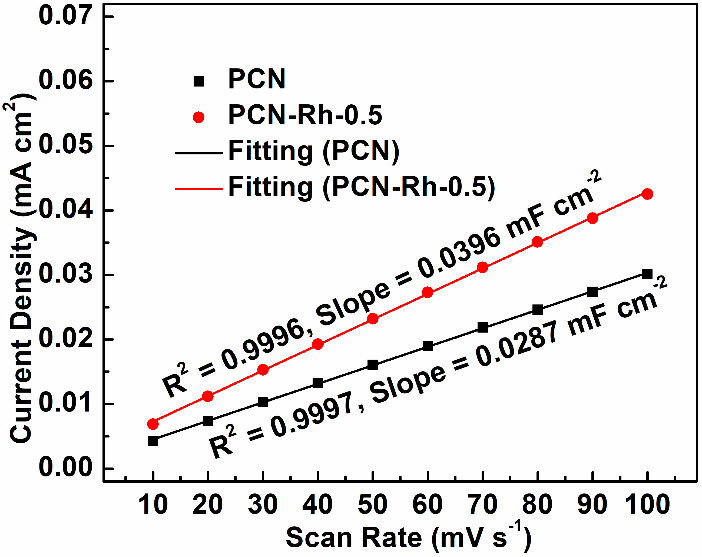


**Figure. S36** Capacitance currents of PCN and PCN-Rh-0.5.


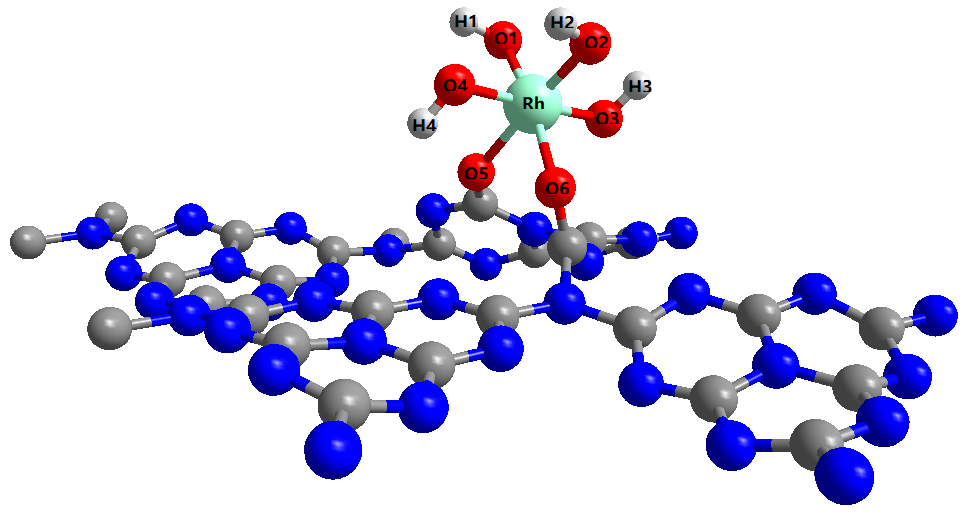


**Figure. S37** Bader charge calculation model of the hydroxylated Rh-SAAs in PCN-Rh


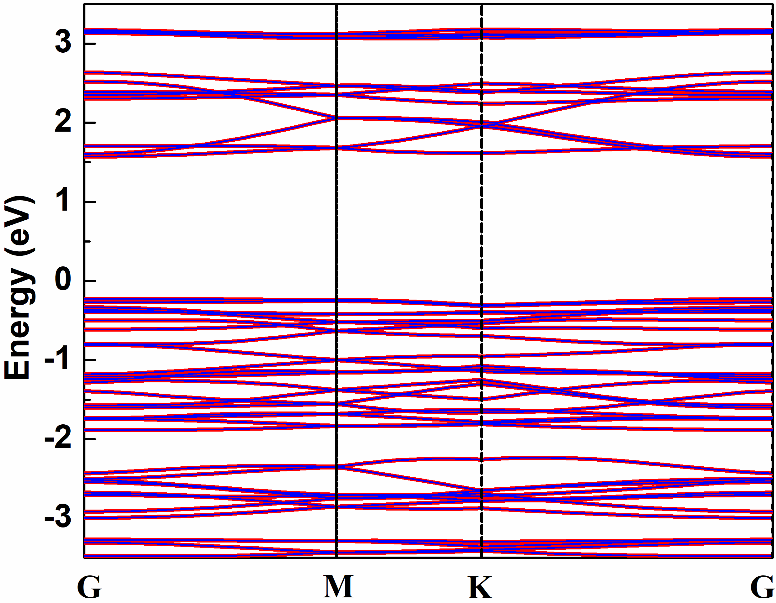


**Figure. S38** Calculated band gap of PCN


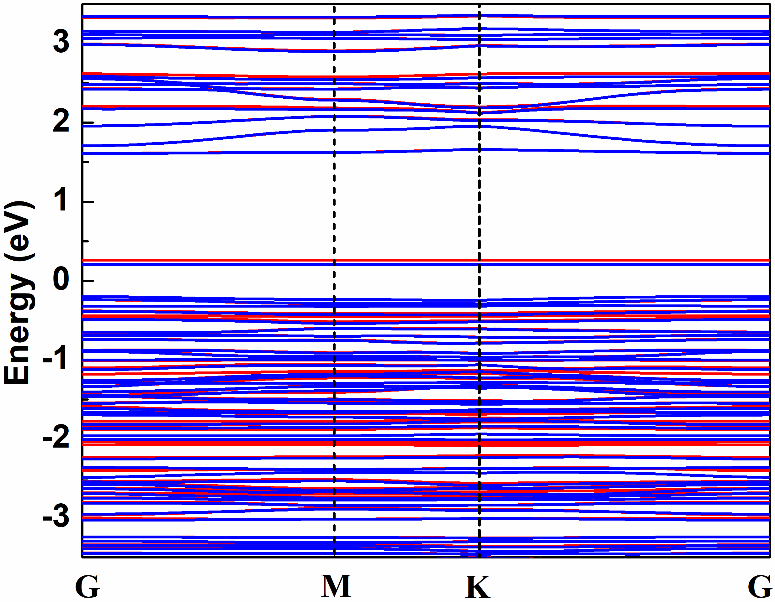


**Figure. S39** Calculated band gap of PCN-Rh


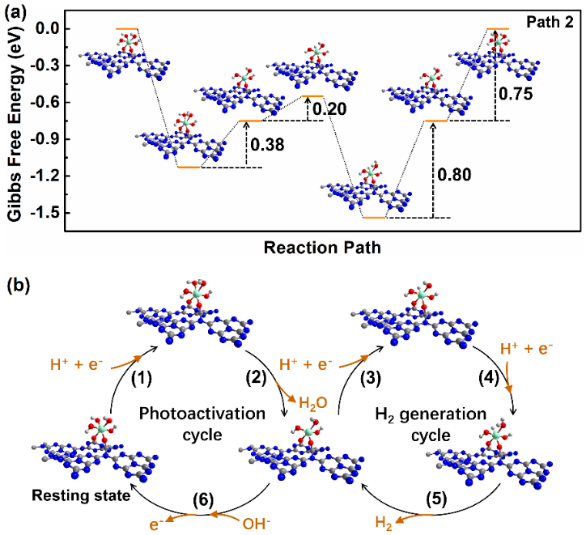


**Figure. S40** (a) free energy diagrams (b) and proposed photocatalytic mechanism for HER on PCN-Rh along Path 2


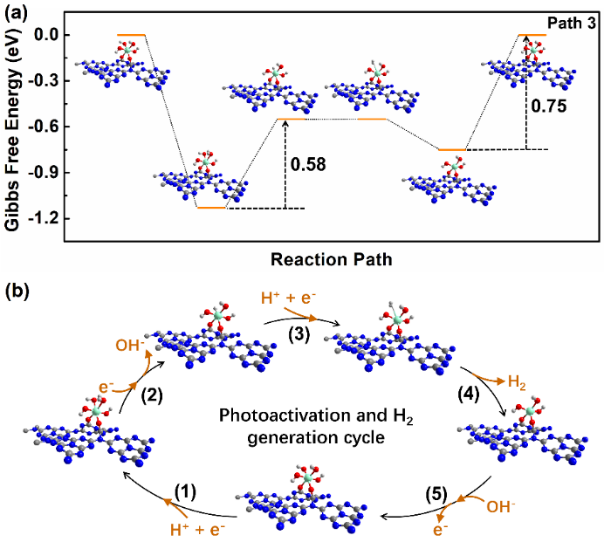


**Figure. S41** (a) free energy diagrams (b) and proposed photocatalytic mechanism for HER on PCN-Rh along Path 3


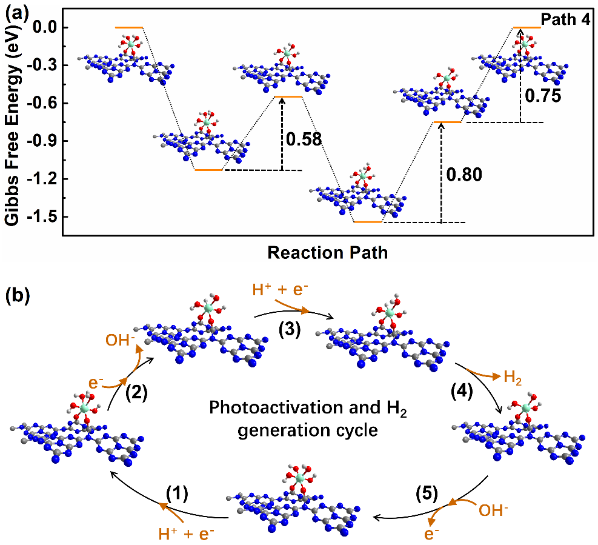


**Figure. S42** (a) free energy diagrams (b) and proposed photocatalytic mechanism for HER on PCN-Rh along Path 4

**Table S1.** The pore size, specific surface area and pore volume of the samples

| Samples | Pore size (nm) | S_BET_ (m^2^ g^-1^) | Pore volume (cm^3^ g^-1^) |
| --- | --- | --- | --- |
| PCN | 15.3 | 66.8 | 0.29 |
| PCN-Rh-0.5 | 9.9 | 66.2 | 0.20 |

**Table S2.** The atomic percentage of different elements in samples obtained from XPS

| Samples | C | N | O | Rh |
| --- | --- | --- | --- | --- |
| PCN | 42.18 | 54.85 | 2.97 | 0 |
| PCN-Rh-0.5 | 44.06 | 52.25 | 3.40 | 0.29 |

**Table S3.** EXAFS fitting parameters

| Materials | Path | N | R (Å) | σ^2^ (Å^2^) | ΔE_0_ (eV) | R factor |
| --- | --- | --- | --- | --- | --- | --- |
| Rh foil | Rh-Rh | 12.0 | 2.68 | 0.003 | -2.2 | 0.0094 |
| Rh_2_O_3_ | Rh-O | 6.0 | 2.02 | 0.002 | 3.5 | 0.0184 |
| PCN-Rh-0.5 | Rh-O | 5.7 | 2.02 | 0.004 | 3.8 | 0.0196 |

**Table S4.** PHE activities of PCN-based single-atom photocatalysts under the same conditions (Sacrificial agent: triethanolamine, Light source: 300 W Xe lamp, Wavelength: λ ≥ 420nm)

| Photocatalyst | Cocatalyst | HER rate (µmol g^−1^ h^−1^) | Running time (h) | Amount of stable H_2_ production (mmol g^−1^) | References |
| --- | --- | --- | --- | --- | --- |
| **PCN-Rh** | **/** | **3409** | **192** | **654.53** | **This work** |
| CCN-SANi | / | 511 | 20 | 10.22 | [8] |
| Pt-MCT-3 | / | 8600 | 17 | 146.20 | [9] |
| Pt_1_-Co_1_/CN | / | 1880 | 16 | 30.08 | [10] |
| Pt-N_3_@MCT | / | 9750 | 16 | 156.00 | [11] |
| PCN-Ru | / | 629.9 | 104 | 65.51 | [12] |
| Ag-N_2_C_2_/CN | / | 1866 | 60 | 111.96 | [13] |
| Co_1_/PCN | / | 182 | 12 | 2.18 | [14] |
| Co_1_Ag_1_-PCN | / | 82 | 20 | 1.64 | [15] |
| FeN_3_P_2_-CN | / | 2668.5 | 8 | 21.35 | [16] |
| In@CNS-5.0 | / | 10110 | 32 | 323.52 | [17] |
| Pd/D_N_-UCN_0.50_ | / | 110.4 | 20 | 2.21 | [18] |
| Cu1/HCNS | / | 3261 | 16 | 52.18 | [19] |
| PtSA-CN | / | 3001 | 16 | 48.02 | [20] |
| SA Pt/g-C_3_N_4_ | / | 22650 | 20 | 453.00 | [21] |
| Ce-SA-C_3_N_4_ | Pt | 33500 | 15 h | 502.50 | [22] |
| W_SA_-CN-PUNS | Pt | 3020 | 12 h | 36.24 | [23] |
| Co-CCN/PTI | Pt | 3538 | 24 h | 84.91 | [24] |
| SA-Cu-CN-620 | Pt | 11230 | 20 h | 224.60 | [25] |
| Co/P/CN-sc | Pt | 3730.4 | 24 h | 89.53 | [26] |
| Co@g-C_3_N_4_ | Pt | 2481 | 16 h | 39.70 | [27] |
| WSA-CN-PUNS | Pt | 3020 | 48 h | 144.96 | [28] |

**Table S5.** PHE activities of high-stability photocatalyst with operation time ≥ 50 h under the same conditions (Sacrificial agent: triethanolamine, Light source: 300 W Xe lamp, Wavelength: λ ≥420nm)

| Photocatalyst | Cocatalyst | HER rate (µmol g^−1^ h^−1^) | Running time (h) | Amount of stable H_2_ production (mmol g^−1^) | References |
| --- | --- | --- | --- | --- | --- |
| **PCN-Rh** | **/** | **3409** | **192** | **654.53** | **This work** |
| ZIS/PAN | **/** | 1836 | 60 | 110.16 | [29] |
| MoC@NGC/ZIS | / | 1012.8 | 60 | 60.77 | [30] |
| NiCoP/g‑C_3_N_4_ | / | 1430 | 60 | 85.80 | [31] |
| Co_9_S_8_/Ag:ZnIn_2_S_4_ | / | 1532.5 | 100 | 153.25 | [32] |
| g-C_3_N_4/_Cu_5_FeS_4_ | / | 27.92 | 100 | 2.79 | [33] |
| RhP_x_/g-C_3_N_4_ | / | 3055.9 | 104 | 317.81 | [34] |
| BP/g-C_3_N_4_ | **/** | 384.17 | 120 | 461.00 | [35] |
| Pt/NP-CN | Pt | 2460 | 60 | 147.6 | [36] |
| CN/BCN | Pt | 3357.1 | 60 | 201.43 | [37] |
| Ti_3_C_2_/g-C_3_N_4_ | Pt | 26.7 | 60 | 1.60 | [38] |
| CoCO_3_/g-C_3_N_4_ | Pt | 717 | 60 | 43.02 | [39] |
| PCN-SA-d | Pt | 8520 | 64 | 545.28 | [40] |
| CN | Pt | 1109.7 | 70 | 77.68 | [41] |
| g-C_3_N_4_/UFRNC_0.02_ | Pt | 1686.4 | 70 | 118.05 | [42] |

**Table S6.** Fitting result from the decay curves of fs-TAS at 483 nm

| Sample | A_1_ (%) | τ_1_ (ps) | A_2_ (%) | τ_2_ (ps) | A_3_ (%) | τ_3_ (ps) |
| --- | --- | --- | --- | --- | --- | --- |
| PCN | 97.55 | 0.17 | 1.29 | 10.66 | 1.16 | 1017.51 |
| PCN-Rh-0.5 | 91.22 | 0.19 | 5.69 | 6.76 | 3.09 | 1001.79 |

**Table S7.** Fitting result from the decay curves of fs-TAS at 725 nm

| Sample | A_1_ (%) | τ_1_ (ps) | A_2_ (%) | τ_2_ (ps) | A_3_ (%) | τ_3_ (ps) |
| --- | --- | --- | --- | --- | --- | --- |
| PCN | 45.72 | 4.18 | 39.23 | 74.28 | 15.05 | 3289.25 |
| PCN-Rh-0.5 | 32.56 | 3.45 | 37.43 | 26.66 | 30.01 | 261.77 |

**Table S8.** TR-PL decay lifetimes of PCN and PCN-Rh-0.5

| Sample | A_1_ | $\tau$_1_ (ns) | A_2_ | $\tau$_2_ (ns) | $\tau$ (ns) |
| --- | --- | --- | --- | --- | --- |
| PCN | 62144.03 | 2.50 | 4447.63 | 8.62 | 3.71 |
| PCN-Rh-0.5 | 39891.18 | 3.44 | 2392.04 | 14.85 | 5.79 |

**Table S9.** Bader charge of the hydroxylated Rh-SAAs in PCN-Rh

| PCN-Rh | H1 | H2 | H3 | H4 | O1 | O2 | O3 | O4 | O5 | H6 | Rh |
| --- | --- | --- | --- | --- | --- | --- | --- | --- | --- | --- | --- |
| Bader charge (e^-^) | 0.61 | 0.60 | 0.59 | 0.60 | -0.89 | -0.91 | -0.90 | -0.97 | -1.09 | -1.04 | 1.69 |

**References**

[1] G. Kresse, J. Furthmüller, Efficiency of ab-initio total energy calculations for metals and semiconductors using a plane-wave basis set. *Computufional Murerials Science*, **1996**, 6, 15-50.

[2] G. Kresse, D. Joubert, From ultrasoft pseudopotentials to the projector augmented-wave method. *Physical Review B*, **1999**, 59, 1758-177.

[3] J. P. Perdew, K. Burke, M. Ernzerhof, Generalized gradient approximation made simple. *Physical Review Letters*, **1996**, 77, 3865-386.

[4] S. Grimme, S. Ehrlich, L. Goerigk, Effect of the damping function in dispersion corrected density functional theory. *Journal of Computational Chemistry*, **2011**, 32, 1456-1465.

[5] V. Wang, N. Xu, J. C. Liu, G. Tang, W. T. Geng, VASPKIT: A user-friendly interface facilitating high-throughput computing and analysis using VASP code. *Computer Physics Communications*, **2021**, 267, 108033.

[6] R. F. W. Bader, A quantum theory of molecular structure and its applications, *Chemical Reviews*, **1991**, 91, 893-928.

[7] W. Tang, E. Sanville, G. Henkelman, A grid-based Bader analysis algorithm without lattice bias, *Journal of Physics: Condensed Matter*, **2009**, 21, 084204.

[8] Z. Lin, Z. Zhang, Y. Wang, Z. Peng, X. Wang, R. Wang, Y. Huang, F. Meng, M. Li, C. Dong, Q. Zhang, L. Gu, S. Shen, Anchoring single nickel atoms on carbon-vacant carbon nitride nanosheets for efficient photocatalytic hydrogen evolution. *Chemical Research in Chinese Universities*, **2022**, 38, 1243-1250.

[9] D. Liu, C. Zhang, J. Shi, Y. Shi, T. T. T. Nga, M. Liu, S. Shen, C. Dong, Pt single atoms toward highly efficient photocatalytic hydrogen production. *Small*, **2024**, 20, 2310289.

[10] M. Yang, J. Mei, Y. Ren, J. Cui, S. Liang, S. Sun, Long-range electron synergy over Pt_1_-Co_1_/CN bimetallic single-atom catalyst in enhancing charge separation for photocatalytic hydrogen production. *Journal of Energy Chemistry*, **2023**, 81, 502-509.

[11] D. Liu, C. Zhang, J. Shi, X. Jin, W. Liu, M. Liu, Y. Chen, L. Guo, Modulating local coordination structure over single Pt atom to optimize adsorption behavior for high-efficiency photocatalytic H_2_ production. *Applied Catalysis B: Environment and Energy*, **2025**, 361, 124655.

[12] C. Li, N. Su, H. Wu, C. Liu, G. Che, Hongjun Dong, Synergies of adjacent sites in atomically dispersed ruthenium toward achieving stable hydrogen evolution. *Inorganic Chemistry*, **2022**, 61, 13453-13461.

[13] X. Jiang, L. Zhang, H. Liu, D. Wu, F. Wu, L. Tian, L. Liu, J. Zou, S. Luo, B. Chen, Silver single atom in carbon nitride catalyst for highly efficient photocatalytic hydrogen evolution. *Angewandte Chemie International Edition*, **2020**, 59, 23112-23116.

[14] Y. Cao, S. Chen, Q. Luo, H. Yan, Y. Lin, W. Liu, L. Cao, J. Lu, J. Yang, T. Yao, S. Wei, Atomic-level insight into optimizing the hydrogen evolution pathway over a Co_1_-N_4_ single-site photocatalyst. *Angewandte Chemie International Edition*, **2017**, 56, 12191-12196.

[15] Y. Liu, Y. Sun, E. Zhao, W. Yang, J. Lin, Q. Zhong, H. Qi, A. Deng, S. Yang, H. Zhang, H. He, S. Liu, Z. Chen, S. Wang, L. Wang, Atomically dispersed silver-cobalt dual-metal sites synergistically promoting photocatalytic hydrogen evolution. *Advanced Functional Materials*, **2023**, 33, 2301840.

[15] X.Cheng, Y. Bi, X. Liu, L.Ji, C. Feng, S. Gao, H. Li, N. Shang, W. Gao, T. Meng, C. Wang, Unraveling the microstructure-property relationship of Fe single-atoms via introducing asymmetric P-coordination for photocatalytic hydrogen evolution. *Advanced Functional Materials*, **2025**, 35, 2413883.

[17] W. Shao, M. Yu, X. Xu, X. Han, Y. Chen, J. Han, G. Wu, W. Xing, Design of a single-atom In–N_3_–S site to modulate exciton behavior in carbon nitride for enhanced photocatalytic performance. *Small*, **2024**, 20, 2306567

[18] M. Ren, X. Zhang, Y. Liu, G. Yang, L. Qin, J. Meng, Y. Guo, Y. Yang, Interlayer palladium-single-atom-coordinated cyano-croup-rich graphitic carbon nitride for enhanced photocatalytic hydrogen production performance. *ACS Catalysis*, **2022**, 12, 5077-5093.

[19] G. Wang, T. Zhang, W. Yu, R. Si, Y. Liu, Z. Zhao, Modulating location of single copper atoms in polymeric carbon nitride for enhanced photoredox catalysis. *ACS Catalysis*, **2020**, 10, 5715−5722

[20] P. Zhou, F. Lv, N. Li, Y. Zhang, Z. Mu, Y. Tang, J. Lai, Y. Chao, M. Luo, F. Lin, J. Zhou, D. Su, S. Guo, Strengthening reactive metal-support interaction to stabilize high-density Pt single atoms on electron-deficient g-C_3_N_4_ for boosting photocatalytic H_2_ production. *Nano Energy*, **2019**, 56, 127-137.

[21] Z. Zeng, Y. Su, X. Quan, W. Choi, G. Zhang, N. Liu, B. Kim, S. Chen, H. Yu, S. Zhang, Single-atom platinum confined by the interlayer nanospace of carbon nitride for efficient photocatalytic hydrogen evolution. *Nano Energy*, **2020**, 69, 104409.

[22] D. Sun, Y. Chen, X. Yu, Y. Yin, G. Tian, Engineering high-coordinated cerium single-atom sites on carbon nitride nanosheets for efficient photocatalytic amine oxidation and water splitting into hydrogen. *Chemical Engineering Journal*, **2023**, 462, 142084.

[23] F. Zhang, J. Zhang, H. Wang, J. Li, H. Liu, X. Jin, X. Wang, G. Zhang, Single tungsten atom steered band-gap engineering for graphitic carbon nitride ultrathin nanosheets boosts visible-light photocatalytic H_2_ evolution. *Chemical Engineering Journal*, **2021**, 424, 130004.

[24] S. Shen, J. Chen, Y. Wang, C. Dong, F. Meng, Q. Zhang, Y. Huangfu, Z. Lin, Y. Huang, Y. Li, M. Li, L. Gu, Boosting photocatalytic hydrogen production by creating isotype heterojunctions and single-atom active sites in highly-crystallized carbon nitride. *Science Bulletin*, **2022**, 67, 520-528.

[25] J. Shen, C. Luo, S. Qiao, Y. Chen, Y. Tang, J. Xu, K. Fu, D. Yuan, H. Tang, H. Zhang, C. Liu, Single-atom Cu channel and N‑vacancy engineering enables efficient charge separation and transfer between C_3_N_4_ interlayers for boosting photocatalytic hydrogen production. *ACS Catalysis*, **2023**, 13, 6280-6288.

[26] W. Li, W. Li, Z. Guo, Y. Song, S. Tang, Y. Ma, X. Xing, Q. Wang, Synthesis of Atomically Thin g‑C_3_N_4_ Nanosheets via Supercritical CO_2_ Doping with Single-Atom Cobalt for Photocatalytic Hydrogen Evolution. *ACS Applied Materials & Interfaces*, **2021**, 13, 52560-52570.

[27] W. Zhang, Y. Fu, Q. Peng, Q. Yao, X. Wang, A. Yu, Z. Chen, Supramolecular preorganization effect to access single cobalt sites for enhanced photocatalytic hydrogen evolution and nitrogen fixation. *Chemical Engineering Journal*, **2020**, 394, 124822.

[28] F. Zhang, J. Zhang, H. Wang, J. Li, H. Liu, X. Jin, X. Wang, G. Zhang, Single tungsten atom steered band-gap engineering for graphitic carbon nitride ultrathin nanosheets boosts visible-light photocatalytic H_2_ evolution. *Chemical Engineering Journal*, **2021**, 424, 130004.

[29] Y. Zhang, L. Niu, Z. Li, T. Yang, Y. Liu, Z. Kang, A recyclable ZnIn2S4/PAN photocatalytic nanofiber membrane for boosting visible light hydrogen evolution in seawater without cocatalyst. *Applied Catalysis B: Environment and Energy*, **2024**, 357, 124300.

[30] S. Liu, X. Zhou, J. Qin, C. Wei, Y. Hu, 3-D nitrogen-doped carbon cage encapsulated ultrasmall MoC nanoparticles for promoting simultaneous ZnIn_2_S_4_ photocatalytic hydrogen generation and organic wastewater degradation. *Journal of Colloid and Interface Science*, **2023**, 635, 59–71.

[31] C. Li, H. Wu, Y. Du, S. Xi, H. Dong, S. Wang, Y. Wang, Mesoporous 3D/2D NiCoP/g‑C_3_N_4_ heterostructure with dual Co−N and Ni−N bonding states for boosting photocatalytic H_2_ production activity and stability. *‌ACS Sustainable Chemistry & Engineering*, **2020**, 8, 12934−12943.

[32] Y. Dou, B. Bai, J. Zheng, X. Wang, Y. Gao, Y. Li, Q. Bu, F. Ding, Y. Sun, Z. Xu, Ag-doped ZnIn_2_S_4_ nanosheets on hollow Co_9_S_8_ polyhedral nanocages as photocatalysts for enhanced hydrogen production and pollutant reduction. *ACS Applied Nano Materials,* **2024**, 7, 5192−5201.

[33] Y. Dou, Y. Gao, B. Bai, J. Zheng, S. Bao, C. Dong, H. Li, Q. Bu, D. Ma, F. Ding, Y. Sun, Z. Xu, Mixed redox-couple-involved bornite phase Cu_5_FeS_4_ as efficient and robust cocatalysts for greatly enhanced visible-light photocatalytic activities. *The Journal of Physical Chemistry C*, **2024**, 128, 14229−14238.

[34] H. Dong, M. Xiao, S. Yu, H. Wu, Y. Wang, J. Sun, G. Chen, C. Li, Insight into the activity and stability of Rh_x_P nano-species supported on g‑C_3_N_4_ for photocatalytic H_2_ production. *ACS Catalysis*, **2020**, 10, 458−462.

[35] Q. Zhang, S. Huang, J. Deng, D. T. Gangadharan, F. Yang, Z. Xu, G. Giorgi, M. Palummo, M. Chaker, D. Ma, Ice-assisted synthesis of black phosphorus nanosheets as a metal-free photocatalyst: 2D/2D heterostructure for broadband H_2_ evolution. *Advanced Functional Materials*, **2019**, 29, 1902486.

[36] C. Li, Y. Ji, Q. Wei, Z. Liu, Y. Wu, L. Chen, D. Han, L. Niu, C. Tao, D. Qin, Defect and electronic structure engineering graphitic carbon nitride with dual-gas-phase reaction for visible-light-driven hydrogen evolution. *ACS Applied Energy Materials*, **2023**, 6, 997−1007.

[37] H. Dong, S. Hong, P. Zhang, S. Yu, Y. Wang, S. Yuan, H. Li, J. Sun, G. Chen, C. Li, Metal-free Z-scheme 2D/2D VdW heterojunction for high-efficiency and durable photocatalytic H_2_ production. *Chemical Engineering Journal* **2020**, 395, 125150.

[38] H. Dong, X. Zhang, Y. Zuo, N. Song, X. Xin, B. Zheng, J. Sun, G. Chen, C. Li, 2D Ti_3_C_2_ as electron harvester anchors on 2D g-C_3_N_4_ to create boundary edge active sites for boosting photocatalytic performance. *Applied Catalysis A: General* **2020**, 590, 117367.

[39] H. Dong, M. Xiao, D. Zhu, Y. Zuo, S. Cheng, Z. Han, C. Li, CoCO_3_ hierarchical structure embedded on g-C_3_N_4_ nanosheets to assemble 3D/2D Z-scheme heterojunction towards efficiently and stably photocatalytic hydrogen production. *International Journal of Hydrogen Energy*, 2021, 46, 32044 -32054.

[40] T. Huo, G. Ba, Q. Deng, F. Yu, G. Wang, H. Li, W. Hou, A dual strategy for synthesizing carbon/defect comodified polymeric carbon nitride porous nanotubes with boosted photocatalytic hydrogen evolution and synchronous contaminant degradation. *Applied Catalysis B: Environmental*, **2021**, 287, 119995

[41] G. Zhao, Y. Cheng, Y. Wu, X. Xu, X. Hao, New 2D Carbon Nitride Organic Materials Synthesis
with Huge-Application Prospects in CN Photocatalyst. *Small* **2018**, 14, 1704138.

[42] H. Che, G. Che, P. Zhou, C. Liu, H. Dong, C. Li, N. Song, C. Li, Nitrogen doped carbon ribbons modified g-C_3_N_4_ for markedly enhanced photocatalytic H_2_-production in visible to near-infrared region. *Chemical Engineering Journal*, **2020**, *382*, 122870.
